# Fast-adapting graph neural network with prior knowledge for drug response prediction across preclinical and clinical data

**DOI:** 10.1016/j.jpha.2025.101386

**Published:** 2025-07-04

**Authors:** Hui Guo, Xiang Lv, Shenghao Li, Daichuan Ma, Yizhou Li, Menglong Li

**Affiliations:** a College of Chemistry, Sichuan University, Chengdu, 610064, China; b Analytical & Testing Center, Sichuan University, Chengdu, 610064, China; c College of Cyber Science and Engineering, Sichuan University, Chengdu, 610064, China

**Keywords:** Few-shot learning, Multi-modal fusion, Explainable artificial intelligence, Drug response prediction

## Abstract

Efficient drug response prediction is crucial for reducing drug development costs and time, but current computational models struggle with limited experimental data and out-of-distribution issues between *in vitro* and *in vivo* settings. To address this, we introduced drug response prediction meta-learner (metaDRP), a novel few-shot learning model designed to enhance predictive accuracy with limited sample sizes across diverse drug-tissue tasks. metaDRP achieves performance comparable to state-of-the-art models in both genomics of drug sensitivity in cancer (GDSC) drug screening and *in vivo* datasets, while effectively mitigating out-of-distribution problems, making it reliable for translating findings from controlled environments to clinical applications. Additionally, metaDRP’s inherent interpretability offers reliable insights into drug mechanisms of action, such as elucidating the pathways and molecular targets of drugs like epothilone B and pemetrexed. This work provides a promising approach to overcoming data scarcity and out-of-distribution challenges in drug response prediction, while promoting the integration of few-shot learning in this field.

## Highlights

• MAML-based bilevel optimization framework to boost drug response prediction model’s generalization in low-sample settings.
• Prior knowledge-driven model with graph neural networks and sparse linear layers for biomolecular network topological information.
• Attention and bio-annotated weights endow model with interpretability to find key drug-related pathways and target genes.

## Graphical abstract

**Figure:**
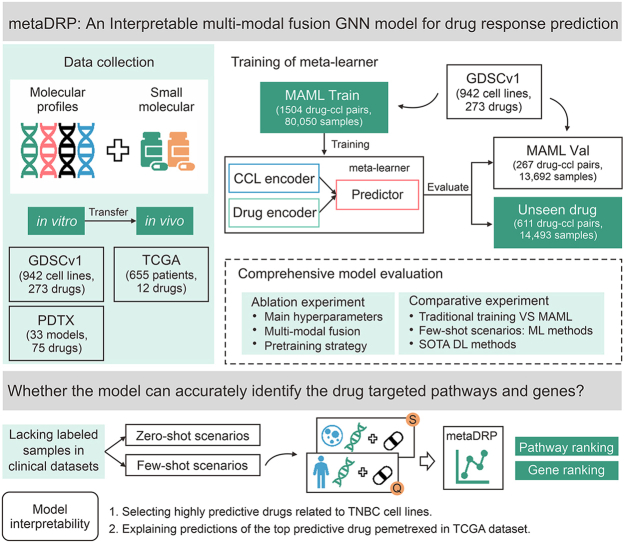

## 1 Introduction

Efficient computational models combined with patient genomic profiles hold promise for personalized drug response prediction (DRP), thereby facilitating pharmacological mechanism research and accelerating drug discovery, which is a critical task in precision oncology. However, developing robust DRP models faces a critical bottleneck: the scarcity of clinical pharmacogenomic data due to prohibitive costs, regulatory constraints, and slow accumulation rates [1]. To circumvent this limitation, cancer cell lines have emerged as indispensable preclinical proxies, recapitulating tumor pathophysiology while enabling high-throughput experimental scalability [2]. Current high-throughput drug screening studies such as Cancer Cell Line Encyclopedia (CCLE) [3,4], Genomics of Drug Sensitivity in Cancer (GDSC) [5], and Cancer Therapeutics Response Portal (CTRP) [6] have systematically profiled hundreds of anticancer compounds across diverse cancer cell lines, generating rich *in vitro* datasets that facilitate the identification of oncogenic drivers and therapeutic response mechanisms [7].

Leveraging these cell line resources, computational DRP models have evolved along two methodological paradigms: single-drug learning and multi-drug learning [8]. Single-drug learning models, including multi-omics late integration method (MOLI) [9], DeepDR [10], drug response variational autoencoder (Dr.VAE) [11], and machine learning methods [12], predict drug-specific responses using cell line molecular profiles. Whereas, these models not only incur high training costs but also fall short in learning the complex associations between multiple drugs and cell lines or tumors. By contrast, multi-drug learning frameworks exemplified by a graph neural network method for cancer drug response prediction (GraphCDR) [13] and twin graph neural networks (GNN) with similarity augmentation (TGSA) [14] adopt a holistic perspective, modeling drug-cell line interactions through shared latent representations. This paradigm not only enhances predictive accuracy by exploiting inter-drug correlations but also enables transductive inference, generalizing to unseen cell lines or drugs through relational graph propagation. This versatility is further amplified by integration of advanced deep learning algorithms such as generative models [15] and reinforcement learning [16], which hold significant potential for expanding their utility in *de novo* drug discovery and repurposing.

Despite these advances, an ongoing challenge lies in the translational gap between preclinical models and human tumors. This mismatch is exacerbated by biological differences across species and tumor microenvironment complexities [7]. Transfer learning strategies have been deployed to mitigate this out-of-distribution (OOD) generalization problem. For instance, Velodrome [17] employs a semi-supervised OOD generalization strategy, introducing alignment loss and consistency loss to enhance the model’s ability of generalization to *in vivo* data. Similarly, single-cell drug response analysis (scDEAL) [18] leverages transfer learning by first training a model on bulk RNA-seq data and subsequently transferring this pre-trained model to scRNA-seq data. This approach enables precise predictions of drug responses at the single-cell level. A context-aware deconfounding autoencoder (CODE-AE) [19] addresses zero-shot learning by proposing a context-aware deconfounding autoencoder which utilizes *in vitro* compound screening data to predict individual patients' responses to new drugs. Meta-learning is also a research paradigm for handling OOD in the domain of DRP. Ma et al. [20] proposed translation of cellular response prediction (TCRP), a few-shot learning framework to improve the transferability of the DRP model from preclinical settings to the clinical environment. Although these methods demonstrate potential, they typically overlook tissue-specific characteristics, whose integration could notably improve the predictive performance for certain drugs [21]. Moreover, data scarcity remains a critical issue when analyzing response patterns of specific drug-tissue combinations, inherently posing a few-shot learning challenge that requires tailored modeling approaches. To tackle these challenges, we employ the model-agnostic meta-learning (MAML) framework [22], a well-established few-shot learning architecture, to train a meta-learner capable of rapid adaptation. By framing tasks around drug-tissue pairs as basic units, our approach explicitly incorporates tissue-specific context during meta-training, enabling efficient fine-tuning with minimal samples of target tasks.

In addition to predictive performance, model interpretability constitutes a pivotal necessity for clinical translation, as opaque ‘black-box’ predictions hinder mechanistic understanding and clinician trust. Recent efforts integrate biological prior knowledge directly into model architectures to enhance intrinsic interpretability. For example, DrugCell [21] aligns neural network layers with gene ontology hierarchies, assigning biological semantics to individual neurons. A sparse and interpretable neural network (SparseGO) [23] employs a pruning strategy to eliminate redundant components from DrugCell, thereby reducing memory consumption. Furthermore, by integrating an external explanation algorithm, deep learning important features (DeepLIFT), SparseGO extends its interpretability through the development of the DeepMoA module, designed to explain the mechanisms of action (MoAs) of drugs. TSGA [14] employs GNNs to explicitly model gene interaction networks, enabling pathway-aware representation learning. Related methodologies, such as drug response predictor and interpreter (DRPreter) [24], also explored similar frameworks for pathway-centric feature extraction. Building upon these foundations, our framework enhances interpretability through two key innovations: (1) hierarchical integration of multi-scale biological features, systematically combining gene-gene interactions and pathway crosstalk; and (2) attention-driven explanatory mechanisms. Specifically, self-attention layers within the cell line encoder dynamically modulate pathway contributions, while cross-attention mechanisms between drug embeddings and pathway activation profiles uncover putative drug-pathway interactions underlying response predictions.

**Fig. 1:**
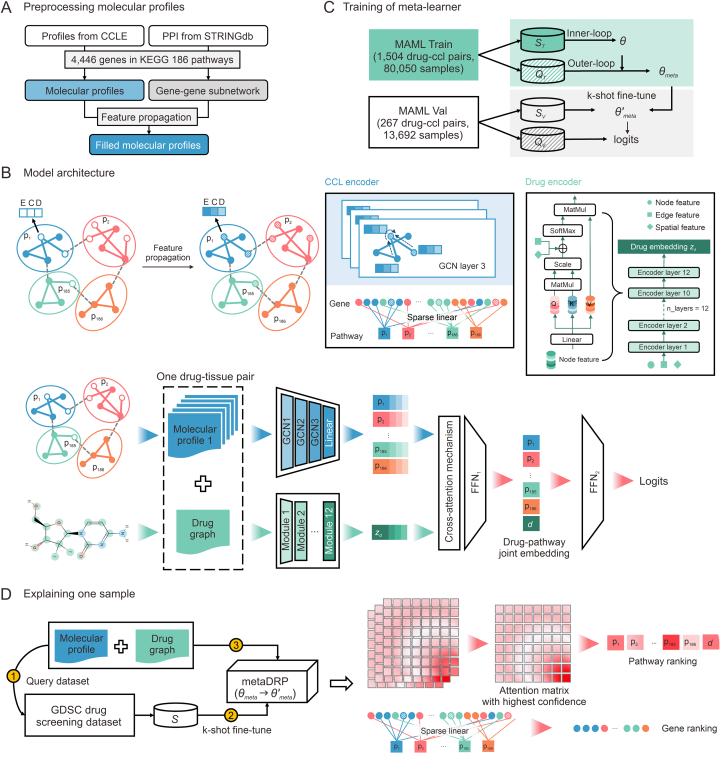
Workflow overview of drug response prediction meta-learner (metaDRP). (A) Molecular profile preprocessing. Raw molecular profiles are subjected to gene subset extraction to generate subnetwork-enriched profiles. Feature propagation is then applied to produce imputed molecular profiles for downstream analysis. (B) Model architecture. The metaDRP model comprises two branches: the cell line branch, which takes molecular profiles as input and generates corresponding pathway embeddings, and the drug branch, which takes a drug graph as input and generates its graph embedding. These are followed by the predictor module, which produces the final predictions. (C) Meta-learner training process. Each drug-tissue task T is randomly split into S and Q subsets. During training, $S_{T}$ is used for the inner-loop updating of task-specific parameters θ, and $Q_{T}$ is for the outer-loop updating of meta-parameters $\theta_{meta}$. For model validation, $S_{V}$ is fine-tunes the model, which is evaluated on $Q_{V}$. (D) Single-sample interpretation pipeline. To interpret a specific sample, metaDRP is fine-tuned on $S_{T}$ (excluding the target sample) from the target drug-tissue task in the Genomics of Drug Sensitivity in Cancer (GDSC) drug screening dataset. The explainer then outputs pathway importance rankings and gene rankings within each pathway. CCLE: Cancer Cell Line Encyclopedia; PPI: protein-protein interaction; KEGG: Kyoto Encyclopedia of Genes and Genomes; MAML Train: model-agnostic meta-learning training dataset; MAML Val: model-agnostic meta-learning validation dataset; GCN: graph convolution network.

In this study, we proposed drug response prediction meta-learner (metaDRP), a few-shot learning model that incorporates biological prior knowledge by leveraging GNNs and attention mechanisms. Our contributions are threefold: First, by constructing meta-training tasks around drug-tissue pairs, we explicitly model tissue-specific context dependencies that are often neglected in previous work. Second, our multi-scale biological aggregation architecture allows for granular interpretation of prediction drivers. Third, through systematic benchmarking across preclinical and clinical cohorts, we demonstrate that metaDRP outperforms baseline and state-of-the-art methods in terms of few-shot adaptability and cross-dataset generalizability. To further understand the decision logic of metaDRP, we employ cross-attention visualization and sparse linear layer analysis. These techniques allow us to pinpoint the critical target genes and pathways that the model uses to make its predictions, providing a transparent and interpretable view of its inner workings. The overview of our workflow is illustrated in Fig. 1.

## 2 Materials and methods

### 2.1 Datasets

#### 2.1.1 Cancer cell line multi-omics molecular profiles

We acquired transcripts per million (TPM) format expression data for protein-coding genes, gene-level copy number variations (CNVs), CRISPR-Cas9 gene effect scores, and DNA methylation profiles within ± 1 kb of transcription start sites (TSS) from the Dependency Map (DepMap) dataset [25]. Annotations of breast cancer cell lines were sourced from Dai et al. [26]. The C2 KEGG_LEGACY subset of canonical pathways, comprising 186 pathways and their corresponding gene subsets was obtained from the Molecular Signatures Database (MSigDB) [27]. Additionally, protein-protein interaction (PPI) data were retrieved from STRINGdb [28]. We retained 4446 protein-coding genes that were represented in Kyoto Encyclopedia of Genes and Genomes (KEGG) (Fig. 1A). The dataset, comprising 1824 human cancer cell line samples, exhibited substantial data gaps across multiple molecular modalities. Specifically, gene expression data was absent in 20.50% of cases, while CNVs were missing in 0.55%. Notably, DNA methylation data was unavailable for over half (54.00%) of the samples, and CRISPR-derived genetic perturbation data was incomplete in 39.97% of instances.

#### 2.1.2 GDSC drug screening dataset

Drug response data for this study was sourced from CellMiner Cross Database (CellMinerCDB) [29], specifically the GDSCv1 dataset, which provides log-transformed half-maximal inhibitory concentration (LN_IC50) values for 942 cell lines and 402 drugs. Cell lines were annotated using OncotreeLineage information based on CCLE metadata, encompassing 31 distinct tissue types. Drug simplified molecular input line entry system (SMILES) codes were retrieved from PubChem, with 282 drugs having valid records. To focus on small molecules, we applied a filtering criterion of SMILES string length <120 characters, retaining 273 drugs. High-molecular-weight compounds like paclitaxel were excluded to prioritize chemically tractable agents.

#### 2.1.3 Drug dataset for pretraining

The Graphormer model was pre-trained using the ZINC 250k dataset [30], a curated collection of drug-like small molecules with SMILES string lengths capped at 120 characters (mean: 35 characters). The dataset includes the water-octanol partition coefficient (logP), a widely recognized metric for quantifying compound lipophilicity, a critical physicochemical property influencing drug absorption, distribution, metabolism, excretion (ADME), toxicity profiles, and pharmacological activity. To optimize computational efficiency while maintaining relevance to drug discovery applications, we focused on utilizing the log*P* values and drug-like molecules for pre-training. Training was conducted with the AdamW optimizer initialized at a learning rate of $5\times 10^{-7}$ and a cosine decay learning rate scheduler with a 600-epoch warmup was employed to gradually adjust the learning rate, balancing convergence speed and model generalization. The model was trained for 10,000 epochs with a batch size of 64.

#### 2.1.4 External dataset

Patient-derived xenograft (PDX) data was sourced from Chen et al. [31], which includes gene expression profiles for 33 PDX models. After excluding drugs with fewer than 15 samples, we constructed a patient-derived tumor xenograft (PDTX) dataset comprising 75 drugs across 20 models. To comprehensively assess the inductive transfer learning ability of metaDRP in clinical settings, we further retrieved gene expression data of The Cancer Genome Atlas (TCGA) patients from Ding et al. [32], which included medication information for 11 small molecule drugs. Identical feature selection and preprocessing pipelines were applied to both PDX and TCGA cohorts to maintain analytical consistency. Predictive performance was assessed by comparing predicted response probabilities with actual treatment outcomes in TCGA patients. Clinical endpoints in the TCGA dataset were defined using response evaluation criteria in solid tumors (RECIST): progressive disease (PD), stable disease (SD), partial response (PR), and complete response (CR). Patients classified with PD/SD were considered non-responders, whereas those with PR/CR were categorized as responders.

#### 2.1.5 Dataset splitting

In the GDSCv1 dataset, the response distributions of the vast majority of drugs (261 out of 273) exhibit significant variation across different tissues (Kruskal-Wallis test, *P* value < 0.01). Employing drug-tissue specific few-shot learning approach would be beneficial to enhance the model’s adaptability to drugs in diverse tissue contexts. To implement this approach, we organized the data according to drug and tissue. To ensure the viability of few-shot learning, we applied a filtering criterion, excluding samples where the number of instances per drug-tissue pair was less than 30. Next, to evaluate the model’s predictive capacity for previously unseen drugs, we then randomly selected 10% of the drugs to form a test set, which was excluded from the training and validation processes of the meta-learner. This test set, referred to as the Unseen Drug dataset, consists of 610 drug-tissue pairs and 14,493 drug response instances. The remaining data were then randomly divided into a training set (85%) and a validation set (15%). The training set, denoted as MAML Train, contains 1503 drug-tissue pairs and 80,050 drug responses. The validation set, labeled as MAML Val, includes 266 drug-tissue pairs and 13,692 drug response instances. A detailed overview of the distribution of each dataset is depicted in Table 1.

**Table 1:** Distribution of all datasets.

Datasets	adrugs	atissues	asamples
MAML Train (model-agnostic meta-learning training dataset)	249	9	80,050
MAML Val (model-agnostic meta-learning validation dataset)	163	9	13,692
Unseen Drug	23	27	14,493
PDTX (patient-derived tumor xenograft)	75	1	976
TCGA (The Cancer Genome Atlas)	11	12	655

MAML Train: model-agnostic meta-learning training dataset; MAML Val: model-agnostic meta-learning validation dataset; PDTX: patient-derived tumor xenograft; TCGA: The Cancer Genome Atlas.

a The number.

### 2.2 Preprocessing methods

#### 2.2.1 Feature propagation

Heterogeneity in sequencing platforms, cohort designs, and sample processing protocols frequently induces missing feature values across genomic datasets. From a graph representation perspective, this corresponds to partially or completely unobserved node attributes. Contrary to conventional approaches that discard samples with incomplete omics profiles, we formulated this challenge as a graph-structured missing value imputation problem. To address this, we implemented the feature propagation algorithm [33], which iteratively reconstructs missing node features through topological diffusion followed by a downstream GNN. Given a cell line $x\in R^{{n\times n}_{o}}$ with adjacency matrix $A\in {\left\{0,1\right\}}^{\left\{n\times n\right\}}$, where *n* is the number of genes and $n_{o}$ is the number of omics types, the computation process for a single iteration is as follows,

(1) $$x\leftarrow \tilde{A}x$$

$\tilde{A}=D^{-\frac{1}{2}}{AD}^{\frac{1}{2}}$, where D is the degree matrix. The number of iterations is set to 40, with empirical validation confirming convergence across all samples through monitoring of L2-norm of feature differences between consecutive iterations (Fig. S1A). Feature propagation also outperformed mean imputation and zero-filling approaches in convergence speed and stability (Fig. S1B).

#### 2.2.2 Construction of drug graph

In this study, we adopted the standardized molecular graph construction framework from the Open Graph Benchmark (OGB) [34], a specialized platform for large-scale benchmarking in graph machine learning to process the SMILES representations. Our implementation rigorously followed OGB’s graph representation paradigm for drug-like molecules: each atom was represented as a graph node characterized by nine fundamental chemical attributes (atomic type, degree, formal charge, hybridization state, chirality, aromaticity, hydrogen count, valence electrons, and ring membership). Chemical bonds were encoded as edges with three critical properties: bond type (single/double/triple/aromatic), conjugation status, and stereochemical configuration. To ensure computational efficiency and alignment with established practices in molecular modeling, we imposed specific constraints on the molecular systems: all drug molecules and drug-like compounds were limited to SMILES strings ≤120 characters for small molecule representation. For the Graphormer architecture implementation, we incorporated spatial encoding through unweighted shortest path distance calculations between atom pairs. This structural descriptor enables synergistic integration of local atomic environments with global molecular topology, effectively bridging the gap between neighborhood-level interactions and whole-molecule spatial relationships. The comprehensive feature engineering strategy, combining these geometric measurements with detailed atomic/bond characteristics, significantly enhanced the graph neural network’s capacity to capture both electronic and steric determinants of molecular properties, thereby improving predictive accuracy in pharmaceutical modeling tasks.

### 2.3 Model architecture

#### 2.3.1 Cell line branch

For a given cell line or tumor sample represented as $G^{c}=\left(V^{c},E^{c},X^{c}\right)$, $V^{c}$ denotes 4,446 genes, $E^{c}$ represents the connections among these genes, and $X^{c}$ represents the node features. We employed a three-layer graph convolution network (GCN) to extract and aggregate gene-level information from the molecular profiles of the cell line or tumor. This configuration enabled nodes to aggregate information from their 3-hop neighbors. By doing so, the receptive field of the GCN is effectively expanded, enabling the model to capture more comprehensive and long-range relationships within the gene network. Residual connections were implemented at each layer of the GCN to alleviate the issue of over-smoothing, which can occur when the GCN aggregates information over multiple layers and may lead to the loss of node-specific information. In contrast to the approach of DRPreter, which necessitates the construction of interaction template networks tailored to cell lines, our approach eschewed network pruning for individual cancers or tissues. Instead, we adopted a standardized network architecture and a consistent subset of cancer-related pathway genes across all cell lines or tumor samples. This strategy is advantageous as it allows the model to adaptively learn tissue- and sample-specific characteristics through transformer-based attention mechanisms while mitigating bias introduction from manual preprocessing steps. After extracting gene-level information, we made a strategic choice to use a sparse linear layer rather than typical graph neural aggregation layers to consolidate gene-level information into pathway-level representations. Each neuron in this layer was sparsely connected to neurons in the subsequent layer that corresponded to a specific KEGG pathway. This sparse connectivity pattern ensured that each pathway representation was derived from a distinct subset of gene features, thereby enforcing pathway-specific information flow and enhancing the model’s interpretability. To further boost the model’s associative learning capabilities, self-attention mechanisms were employed to integrate pathway-level features, enabling the model to weigh different pathway features according to their importance and ultimately yield pathway-level representations $Z_{i}^{c}\in R^{186\times n_{e}}$ for the cell line i, where $n_{e}$ is the embedding dimension. The parameters of the cell line branch encoder are configured as $W_{c}$.

#### 2.3.2 Drug branch

Graphormer is a graph transformer model renowned for its inherent interpretability, and has demonstrated remarkable performance in a wide array of graph-related tasks [35]. This model ingeniously integrates the potent representational capabilities of the transformer-based model into graph-structured data by introducing three innovative structural encodings: centrality encoding, spatial encoding, and edge encoding. These enhancements enable the model’s self-attention layer to more effectively identify and prioritize ‘important’ nodes or node pairs, thereby facilitating more precise allocation of attention weights in subsequent layers. In our study, we employed the SLIM version of the Graphormer architecture and pretrained the model on the ZINC 250k dataset. Specific parameters are detailed in Table S1. Given a molecular graph $G^{d}$,we derived the representation $z_{j}^{d}\in R^{n_{e}}$ for drug j from the drug branch with the parameters set as $W_{d}$.

#### 2.3.3 Predictor

The predictor was composed of two components: the fusion of multi-modal information between the cell line/tumor and the drug, and the feedforward prediction module, as illustrated in Fig. 1B. We first concatenated the cell line embedding $Z_{i}^{c}\in R^{186\times n_{e}}$ and the drug embedding $z_{j}^{d}\in R^{n_{e}}$ into $H_{i,j}\in R^{{187\times n}_{e}}$. To achieve effective multi-modal information fusion, we employed a cross-attention mechanism. The cross-attention calculation was carried out in the following steps:

(2) $$H_{i,j}=\text{concat}\left(Z_{i}^{c},z_{j}^{d}\right)$$

(3) $$Q_{i,j},K_{i,j},V_{i,j}=\text{Linear}\left(H_{i,j}\right)$$

(4) $${H_{i,j}}^{\prime }={\left(\text{softmax}\left(\frac{{Q_{i,j}K_{i,j}}^{T}}{\sqrt{d_{k}}}\right)V_{i,j}\right)}^{T}$$

where Linear is represented for a linear transformation, and $d_{k}$ is the embedding size of $K_{i,j}$.

After obtaining the attention-weighted output ${H_{i,j}}^{\prime }$, We then used a feedforward network ${\text{FNN}}_{1}$ to further process this information. The output of FFN_1_ was a joint representation $z_{i,j}^{cd}\in R^{n_{e}}$. Subsequently, the drug response predictions were obtained through a second feedforward network ${\text{FNN}}_{2}$. The parameters of the entire predictor are established as $W_{p}$.

### 2.4 Model training and prediction

To improve the molecular representation quality of our drug encoder, we implemented a two-stage training paradigm. The drug encoder was first pretrained on the ZINC 250k dataset to learn fundamental molecular pattern recognition. This pretrained encoder was subsequently integrated into our meta-learning architecture during initial meta-training phases. Our implementation of MAML incorporated a dual-loop optimization mechanism to handle heterogeneous tissue-specific response patterns: (1) inner-loop adaptation: task-specific rapid fine-tuning using support samples from meta-tasks; (2) outer-loop meta-optimization: cross-task generalization based on query samples. We trained the meta-learner using the MAML Train dataset, concurrently evaluating performance on the MAML Val dataset (Fig. 1C). The complete meta-training protocol (Table 2) followed these specifications. Each meta-task represented a unique drug-tissue interaction scenario, denoted by a drug-tissue pair ${Τ}_{i}$ and was randomly selected to include 30 samples. Of these, 10 were designated as support samples ($S_{i}$) for inner-loop weight updating and 20 as query samples ($Q_{i}$) for outer-loop weight optimization. The inner-loop learning rate was fixed at 0.001, and the Adam optimizer was employed for updating meta-learner’s parameters $\theta_{meta}$ during outer-loop optimization. To mitigate the risk of overfitting, dropout layers were incorporated into all model trainable components, and an early stopping criterion was implemented, halting training after 50 consecutive epochs with non-decreasing validation loss. All training parameters remained consistent across ablation studies to ensure fair comparison (Table S1). For few-shot predictions on test tasks, k samples were randomly chosen from given tasks for fine-tuning the well-trained meta-learner (k-shot), followed by predictions on the remaining samples. Detailed updating calculations were as follows:

**Table 2:** The training algorithm of model-agnostic meta-learning (MAML).

**Algorithm 1.** Training of meta-learner.	
1:	initialize $\theta_{meta}=\theta =\left\{W_{c},W_{d},W_{p}\right\}$ randomly, if a pretrained drug encoder is available, take its parameter as $W_{d}$.
2:	**while** not done **do**:
3:	sample a batch of drug-tissue tasks ${Τ}_{i}$
4:	**for** all ${Τ}_{i}$**do**
5:	sample support set $S_{i}$ and query set $Q_{i}$ from ${Τ}_{i}$
6:	update θ according to Eqs. (5), (6)
7:	**end for**
8:	update $\theta_{meta}$ according to Eqs. (7), (8), (9)
9:	**end while**

Single updating step in the inner loop,

(5) $$L_{inner}^{i}=\frac{1}{\left|S_{i}\right|}\sum_{j=1}^{\left|S_{i}\right|}\text{MAE}\left(f_{\theta }\left(x_{S_{i}}^{j}\right),y_{S_{i}}^{j}\right)$$

(6) $$\theta \leftarrow \theta -\alpha \nabla L_{inner}^{i}$$

Single updating step in the outer loop,

(7) $$L_{outer}^{i}=\frac{1}{\left|Q_{i}\right|}\sum_{j=1}^{\left|Q_{i}\right|}\text{MAE}\left(f_{\theta }\left(x_{Q_{i}}^{j}\right),y_{Q_{i}}^{j}\right)$$

(8) $$L_{outer}=\frac{1}{T}\sum_{i=1}^{T}L_{outer}^{i}$$

(9) $$\theta_{meta}\leftarrow \theta_{meta}-\beta \nabla L_{outer}$$

### 2.5 Baseline models

We systematically evaluated conventional machine learning approaches in low-data scenarios by benchmarking three distinct algorithms: ridge regression (Ridge), random forest (RF), and support vector machine (SVM). These algorithms were implemented using the *scikit-learn* package with hyperparameter optimization via grid search (Table S2). Due to challenges in integrating multi-modal inputs for drugs and cell lines, all ML methods uniformly utilized cell line gene expression profiles as input features, with separate models trained per drug-tissue pair. Our evaluation protocol imposed strict quality controls: (1) only tasks containing ≥7 samples were included to ensure statistical reliability; (2) identical sample splits were maintained across compared methods; (3) performance metrics were calculated exclusively on hold-out samples not involved in model training. The benchmark analysis extended to state-of-the-art deep learning approaches (TGSA, MOLI, DrugCell, and PaccMann) using published drug response predictions from Shen et al. [8], and comparing the drug predictive performance on the GDSCv1 dataset (231 anticancer compounds). For these methods, 5-fold cross-validation results were used to assess drug-level performance, whereas metaDRP’s 10-shot performance on all query samples was reported. To isolate the benefits of meta-learning, we implemented two ablation models: (1) conventional deep learning architecture trained on MAML Train data without task-specific fine-tuning (DRP^woFT^); (2) identical architecture fine-tuned on target task samples (DRP^wFT^). Both variants underwent identical hyperparameter optimization. To evaluate the inductive transfer learning ability of the metaDRP based on TCGA cohort, three metrics were calculated between responder/non-responder groups: effect size, *p* value (Mann-Whitney U), and area under the receiver operating characteristic curve (ROC_AUC). Results were compared against state-of-the-art deep learning benchmarks.

### 2.6 Model explainer

The implementation of multi-head attention mechanisms is a common practice aimed at enhancing the model’s representation learning capabilities. Nevertheless, this process presents interpretative challenges for attention scores due to multi-layer non-linear transformation. To address this, we referred to the methods described in Voita et al. [36] for handling these scores and calculating confidence scores to pinpoint critical layers and heads for detailed analysis. We randomly selected support samples for few-shot fine-tuning and repeated the process 10 times. For each repetition, we first calculated the head’s confidence scores, then average these scores to obtain the final attention matrix for analysis. We investigated the model’s intrinsic interpretability from two viewpoints. First, we prioritized pathways through the pathway-drug cross-attention module and examined whether the target pathways were ranked among the top 10. Second, we identified key gene subsets for pathways based on the weights of sparse linear aggregation layer of the cell line branch and then investigated whether the associated target genes were placed within the top 25% of their respective pathways.

### 2.7 Evaluation metrics

We reported common metrics for regression tasks, including mean square error (MSE), mean absolute error (MAE), pearson correlation coefficient (PCC), and Spearman’s rank correlation coefficient (SCC). To comprehensively assess the model’s predictive capabilities from multiple aspects, we established four evaluation scenarios: (1) Overall performance across all drugs and tissues (metrics^a^). We aggregated the logits from all model predictions and compared them with the actual labels. This analysis offered a broad assessment of the model’s overall accuracy, providing an understanding of how well the model performs when considering all drugs and tissues collectively; (2) Drug-level performance (metrics^d^). For each individual drug, we calculated the performance metrics across all tissues to assess the model’s predictive power for individual drugs and identify any drug-specific biases; (3) Tissue-level performance (metrics^t^). We calculated the performance metrics for each tissue across all drugs, to evaluate the model’s effectiveness in predicting outcomes for various tissues, highlighting potential tissue-specific strengths or weaknesses; (4) Drug-tissue level average performance (metrics^dt^). We reported the average metrics for each drug-tissue pair, along with their 95% confidence intervals (CI) to provide insights into the model’s precision at the intersection of drug and tissue types. SCC^dt^ was used as the criterion for selecting the best model, with an SCC value greater than 0.5 being indicative of high predictive performance. These scenarios are designed to ensure that the model’s performance is not only accurate on average but also consistently reliable across varied biological contexts.

### 2.8 Visualization of drug embeddings

We visualized the drug embeddings generated by the drug encoder, both from the pretrained and non-pretrained models. These visualizations were then combined with k-Means analysis to explore the clustering patterns of these embeddings. Given the substantial correlation between drug functions and structures, we conducted an additional analysis using Fisher’s exact test to investigate the relationship between the k-Means clusters and the respective drug MoAs. *P* value < 0.05 was considered a significant difference (∗*P* value < 0.05, ^∗∗^*P* value < 0.01, ^∗∗∗^*P* value < 0.001).

### 2.9 Analysis of correlation between the model’s predictive performance and drug functions

To systematically investigate the factors influencing predictive performance across diverse pharmacological contexts, we conducted a multi-scale analysis integrating metaDRP’s 10-shot predictions from both MAML Train and MAML Val datasets. Compounds were stratified into predictable (P, top 25%) and unpredictable (U, bottom 25%) categories based on the quantiles of drug-level metrics SCC^d^. Response distribution characteristics were quantified using three metrics: standard deviation (STD), bimodality coefficient (BC), density coverage (DC) rate of LN_IC50. For drug-tissue interaction analysis, we curated 1608 tasks with mechanistically annotated targets (22 MoAs × 9 tissues), excluding abl signaling-targeted drugs due to limited sample size (*n* < 5). A robust median regression model was applied:

(10) $$\text{Median}\left({\text{SCC}}^{\text{dt}}\right)=\beta_{0}+\beta_{1}D_{\text{MoA}}+\beta_{2}D_{\text{Tissue}}+\epsilon$$

where $D_{\text{MoA}}$ and $D_{\text{Tissue}}$ represent binary indicator for drug MoAs and tissue type, respectively. Statistical significance was assessed through permutation testing (1000 iterations) followed by Benjamini-Hochberg false discovery rate (FDR) correction (α=0.05). Bootstrap resampling (1000 replicates) provided 95% confidence intervals for effect sizes. This analysis revealed distinct predictability patterns shaped by drug response distribution characteristics, target pathway specificity and tissue microenvironment interactions.

## 3 Results

### 3.1 The metaDRP model outperforms the baseline methods on MAML Val dataset

**Fig. 2:**
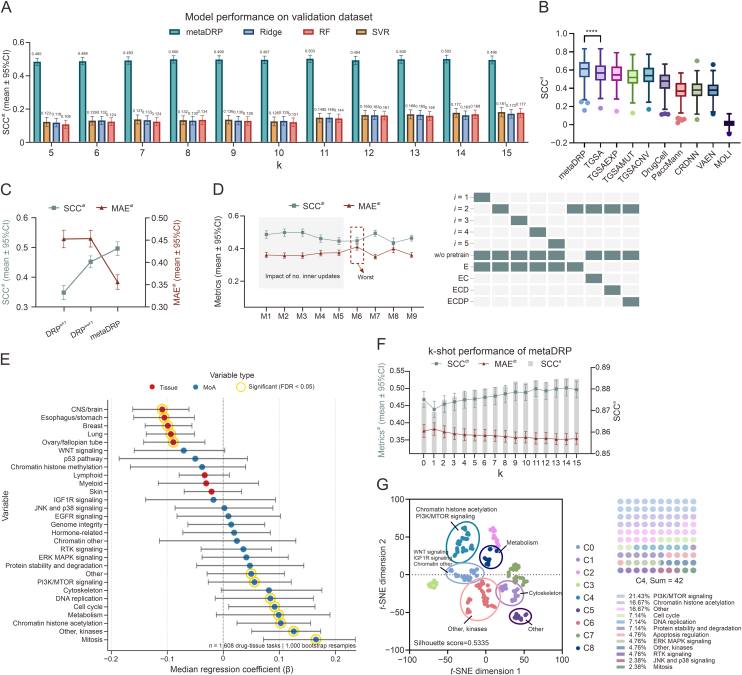
Comprehensive performance evaluation of drug response prediction meta-learner (metaDRP) on the genomics of drug sensitivity in cancer (GDSC) dataset. (A) Model performance comparison among metaDRP, ridge regression (Ridge), random forest (RF), and support vector machine (SVR) in terms of drug-tissue Spearman’s rank correlation coefficient (SCC^dt^) on model-agnostic meta-learning validation dataset (MAML Val), with results reported as mean ± 95% confidence intervals (CI). (B) Drug-level prediction benchmarking. Comparative performance of metaDRP (10-shot) against other deep learning-based DRP models on 131 drugs measured by drug-level Spearman correlation (SCC^d^). Statistical significance is denoted as: ^∗∗∗∗^*P* < 0.0001. (C) Meta-learning vs. conventional training. Comparison of DRP (DRP^wFT^: with fine-tuning; DRP^woFT^: without fine-tuning) and metaDRP on drug-tissue performance metrics SCC^dt^ and mean absolute error (MAE^dt^). (D) Hyperparameter optimization analysis demonstrating the impact of inner-loop update frequency, pretraining on drug encoder and multi-omics fusion, where ‘i’ denotes the number of inner loop updates, ‘w/o pretrain’ signifies the absence of pretrained drug encoder, and ‘ECDP’ corresponds to multi-omics data types (E: gene expression, C: copy number variations, D: DNA methylation, P: CRISPR-Cas9 gene effects). Darker shades indicate selected configurations. (E) Mechanistic and tissue-specific associations. Median regression analysis of 1,608 drug-tissue tasks reveals significant positive/negative associations between model predictive performance and pharmacological mechanisms of actions (MoAs; red)/tissue types (blue). Statistical significance was determined through 1,000-iteration permutation testing with Benjamini-Hochberg false discovery rate (FDR) correction (α=0.05). Shaded regions represent bootstrap-estimated 95%CI from 1,000 bootstrap resamples. (F) Few-shot learning performance. Performance metrics (SCC^dt^ and MAE^dt^) under varying k-shot conditions was depicted as line graphs, and the overall performance SCC^a^ was presented in a bar chart. (G) Drug embedding clustering analysis. *t*-SNE visualization of pretrained drug molecule embeddings was colored by k-means clusters (C0–C8). Significant enrichments (Fisher’s exact test, *P* value < 0.05) of drug MoAs are marked. Inset: C4 cluster shows significant enrichment of PI3K/mTOR signaling-targeted drugs.

We initially compared metaDRP against four ML methods on the MAML Val dataset. All methods demonstrated consistent improvement in predictive performance with increasing numbers of fine-tuning/training samples (Fig. 2A, and Table S3). Notably, metaDRP achieved superior few-shot learning capabilities compared to ML baselines in this low-data scenarios, suggesting its superior adaptability to limited data. Despite the high overall predictive accuracy of all methods with SCC^a^ greater than 0.8 (Table S3), the performance metrics deteriorated when evaluated at the drug or tissue level. This suggests challenges in capturing nuanced response patterns specific to individual drugs or tissues, even with high-quality global predictions. Subsequently, we employed the trained meta-learner to conduct 10-shot learning experiments on all drug-tissue tasks in the GDSCv1 dataset. We compared the model’s drug level performance (SCC^d^) against previously reported deep learning models (Fig. 2B). The metaDRP model demonstrated superior performance across 231 drugs compared to other tested methods. A one-tailed paired *t*-test confirmed significantly higher mean prediction SCC^d^ of metaDRP than that of the previously reported best-performing TGSA (*P* value < 0.0001, A−B = 0.0316, A: metaDRP, B: TGSA), underscoring its broader applicability. Direct comparison of MAML-based metaDRP with conventional training methods (DRP) revealed significant performance gains despite identical architecture and fine-tuning protocols. The metaDRP outperformed DRP across all evaluation metrics, highlighting the critical role of MAML’s dual-loop optimization in improving cross-task generalization (Fig. 2C).

### 3.2 Advancing drug response prediction through multi-omics data integration

Building upon our previous work in GCN-based multi-omics fusion, we systematically evaluated metaDRP’s capacity for integrative omics processing. Our analysis revealed that the model integrated gene expression data and CNVs (M7) achieved slightly superior performance (MAE^dt^: 0.3498±0.0142) compared to the expression-only baseline M2 (MAE^dt^: 0.3567±0.0169), with 52.98% of compounds showing enhanced predictive accuracy (Fig. 2D, and Table S4). This demonstrates the significant role of CNVs in capturing drug response patterns (Text S1). Conversely, models integrating more extensive omics data (M8 and M9) paradoxically degraded performance, likely due to high missingness rates in omics data compromising imputation fidelity and inadequate modeling of relationships between DNA methylation or gene dependency and drug sensitivity (Fig. 2D, and Table S4). We further implemented the feature zeroing approach during the model testing phase to evaluate the importance of each omics type, quantifying the significance of different omics features by monitoring alterations in the SCC^d^. Experimental results demonstrate that CNV constitutes the most informative feature, followed by gene expression profiles. The exclusion of these two feature sets leads to a substantial decrement in the model’s predictive accuracy. Conversely, the removal of DNA methylation and CRISPR screening data exhibits negligible effects on model performance, indicating that the model places relatively low reliance on information from these two feature types during the decision-making process (Table S5). Notably, all models showed elevated SCC values in cell cycle-targeted therapies, suggesting conserved pathway-specific omics signatures (Fig. S2A). Specific therapeutic contexts revealed complementary strengths: M7 demonstrated unique predictive capabilities for 10 drugs, whereas M2, M8 and M9 excelled for 28, 14 and 4 drugs, respectively (Fig. S2B, and Tables S6–9). These findings highlight the potential of multi-omics integration in improving drug response prediction performance, underscoring the critical role of genetic and epigenetic features in personalized medicine [37].

### 3.3 Associations between M2’s predictive performance and drug MoAs/tissue biology

Our systematic analysis of M2’s predictive performance revealed distinct associations with pharmacological mechanisms and tissue biology (Fig. 2E). Tissue-specific effect sizes demonstrated significant negative correlations in central nervous system (CNS)/brain, esophagus/stomach, breast, lung, and ovary/fallopian tube tissues (FDR-adjusted *P* value < 0.01), suggesting gene expression profiles alone may insufficiently capture tissue microenvironment complexities and critical physiological barriers (e.g., blood-brain barrier permeability determinants). Conversely, MoA analysis identified strong positive predictability associations for mitosis inhibitors, other kinase-targeted agents, cell cycle regulators, chromatin histone acetylators, and metabolic pathway modulators. The superior performance on mitosis-targeted compounds (e.g., microtubule inhibitors like epothilone B) aligns with our mechanistic validation in Section 3.7, where attention mapping confirmed the model’s capacity to identify tubulin-binding domains critical for drug activity. These significant positive associations between pharmacological MoAs and model predictability confirm the framework’s capacity to extract mechanistically relevant drug features. Conversely, the observed negative tissue effects reveal critical gaps in microenvironmental representation that require further investigation.

### 3.4 Selecting the optimal hyperparamers of metaDRP

The metaDRP model was trained using the MAML Train dataset, with hyperparameter configuration optimized based on the MAML Validation dataset. The primary criterion for selecting the optimal model was SCC^dt^. We systematically evaluated the impact of inner-loop update frequency (1–5 iterations) on model performance, which directly affects computational efficiency. We observed that the performance with a single update was satisfactory (SCC^dt^: 0.4860±0.0224), and there was a notable improvement with two updates (SCC^dt^: 0.4972±0.0226). However, three updates showed minimal gain (SCC^dt^: 0.4978±0.0226), and further updates paradoxically decreased performance (Fig. 2D, and Table S4). These results demonstrated that two inner-loop updates provided the optimal balance between computational efficiency and efficacy.

We then evaluated the influence of the number of support samples used by the meta-learner during the prediction phase. The zero-shot configuration, which requires no fine-tuning, remarkably outperformed the one-shot scenario. Specifically, it exhibited high predictive performance (SCC^dt^ > 0.5) in 49.43% (132 out of 265) of drug-tissue pairs. This finding underscores that the zero-shot approach not only delivers high predictive performance but also shows inherent robustness right from the initialization stage, making it highly suitable for predicting outcomes of unknown drug-tissue pairs and offering valuable predictive insights in scenarios where prior data is scarce. Incorporating more than one sample in few-shot learning further enhances the model’s generalization ability (Fig. 2F). As the number of fine-tuning samples increased, there was a progressive improvement in both the average and overall performance and the few-shot models contributed to a total improvement of about 4% compared to the zero-shot models.

### 3.5 Pretrained drug encoder enhances the model’s capacity of drug representation

Pretraining the drug branch exerted a significant impact on model performance. The GDSCv1 experiment was restricted to 273 drugs, potentially limiting the representation and generalization capabilities of complex deep learning models due to insufficient samples. The metaDRP with a pretrained drug branch (M2) notably outperformed non-pretrained variant (M6), which achieved the poorest performance (SCC^dt^: 0.4466 ± 0.0231) under identical conditions (Fig. 2D). Notably, the pretrained drug encoder demonstrated enhanced discriminatory power by effectively differentiating drug types, as drugs targeting the PI3K/MTOR signaling pathway predominantly clustered in cluster C4, and metabolism-targeted drugs preferentially grouped in cluster C8 (Fig. 2G). In contrast, non-pretrained drug embeddings showed poor clustering quality and non-significant correlation with drug MoAs (Fig. S2C). These findings highlight that effective pretraining significantly enhances the model’s capacity to learn discriminative drug representations, thereby improving its discriminatory power for downstream predictive tasks.

### 3.6 Evaluating metaDRP’s predictive performance and adaptability for unseen drugs across few-shot learning scenarios

**Fig. 3:**
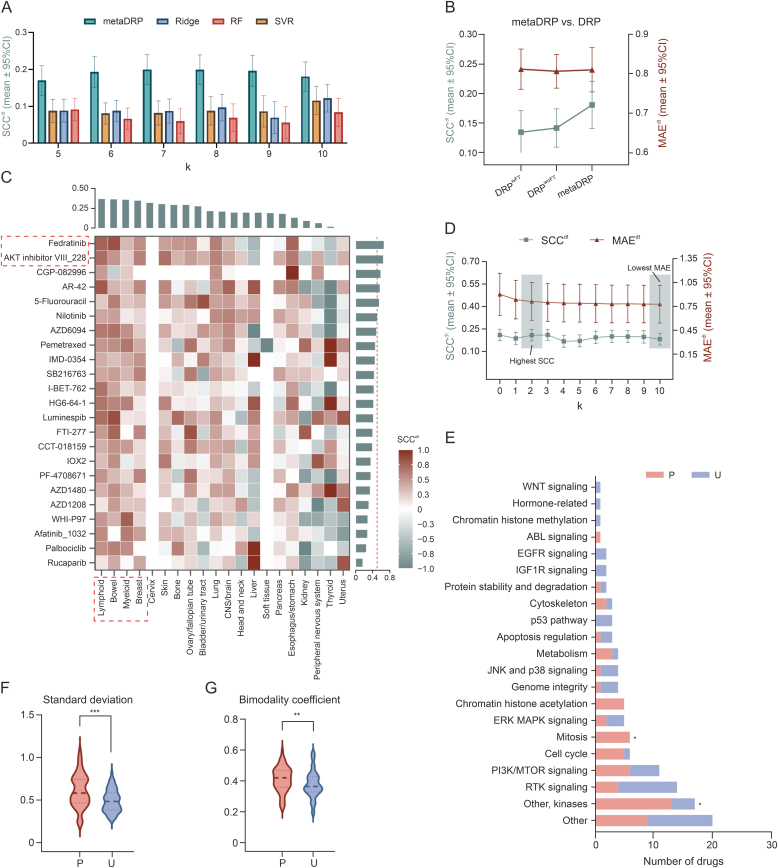
Performance evaluation on Unseen Drug dataset. (A) Model performance comparison among drug response prediction meta-learner (metaDRP), ridge regression (Ridge), random forest (RF), and support vector machine (SVR) in terms of drug-tissue Spearman’s rank correlation coefficient (SCC^dt^) on Unseen Drug, with results reported as mean ± 95% confidence intervals (CI). (B) Predictive performance of metaDRP vs. DRP (DRP^wFT^: with fine-tuning; DRP^woFT^: without fine-tuning) for unseen drugs, evaluated using SCC^dt^ and mean absolute error (MAE^dt^). (C) 10-shot prediction heatmap. Heatmap visualizes metaDRP’s drug-tissue predictive performance (SCC^dt^) for unseen drugs. Top bar chart displays tissue-level Spearman’s rank correlation coefficient (SCC^t^), while right bar chart shows drug-level Spearman’s rank correlation coefficient (SCC^d^). (D) Performance comparison of metaDRP under 0-shot and few-shot learning scenarios for unseen drugs. (E) Drug distribution by mechanisms of action (MoA), with ‘∗’ denoting a Fisher’s exact test *P* value < 0.05 for pathway enrichment analysis. P: predictable class; U: unpredictable class. (F, G) Response distribution analysis. Violin plots show the distribution of standard deviation (F) and bimodality coefficient (G) for predictable (P-class) and unpredictable (U-class) drugs from MAML Train and MAML Val dataset. Statistical significance is denoted as: ^∗∗∗^*P* < 0.001, ^∗∗^f*P* < 0.01.

We assessed the metaDRP model’s ability to predict responses of unseen drugs using the Unseen Drug dataset. Comparative analysis across varying k-shot/training sample sizes at the drug-tissue level demonstrated metaDRP’s superior performance over baseline methods (SVR, Ridge, RF, and DRP) indicating its inferential capability for novel compounds (Figs. 3A and B). Specifically, metaDRP achieved high-confidence predictions for 6 out of 23 drugs in 10-shot scenarios, with significant improvements over 0-shot prediction for specific compounds like fedratinib (SCC^d^ increases from 0.6191 to 0.6577), and AKT inhibitor VIII (SCC^d^ rises from 0.5831 to 0.6418). At the tissue level, metaDRP’s predictions for unseen drugs were less accurate, with most SCC^t^ scores below 0.5 (Fig. 3C). While the MAE for few-shot scenarios decreased with increasing fine-tuning samples, suggesting quick adaptation to new tasks, a weak correlation between MAE and SCC indicates that lower error rates do not necessarily correlate with more accurate drug response predictions (Fig. 3D, and Table S10).

Pathway enrichment analysis identified certain pathways (other, kinases, and mitosis) significantly associated with drug predictability (Fig. 3E). Analysis of drug response pattern-predictability correlations revealed significant differences in STD and BC between P-class and U-class drugs, indicating that drugs with high response variability tend to be more predictable (Figs. 3F, 3G, and S2D). Regard to unseen drugs, the top 10 predictable drugs exhibited higher STD and BC compared to the bottom 10 (Fig. S2E). These results suggest that there is a certain relationship between drug predictability and their functions as well as response distributions. Drugs with high STD and BC may be more predictable.

### 3.7 Comprehensive evaluation of metaDRP’s predictive performance in preclinical and clinical settings

**Fig. 4:**
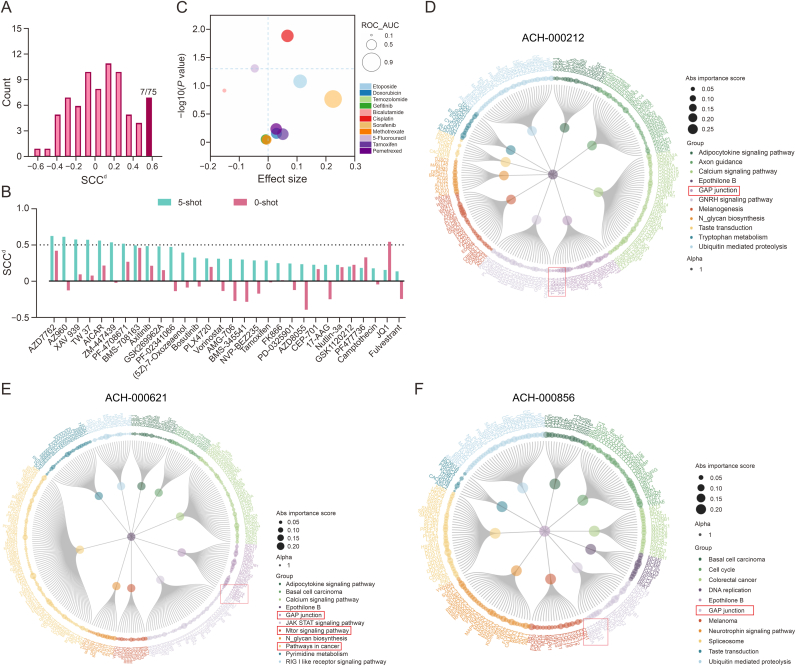
Model performance on external dataset patient-derived tumor xenograft dataset (PDTX) and The Cancer Genome Atlas dataset (TCGA). (A) Histogram displaying the model drug-level Spearman’s rank correlation coefficient (SCC^d^) for metaDRP on PDTX drugs. (B) Performance of metaDRP’s 5-shot and 0-shot predictions for the drug screening cohort of the PDTX dataset are shown in terms of SCC^d^. (C) Results of metaDRP’s 0-shot predictions on 11 drugs from TCGA dataset, with metrics including area under the receiver operating characteristic curve (ROC_AUC, circle size), effect size (axis *x*), and *P* value (axis *y*). (D–F) Explanation of ACH-000212 (D), ACH-000621 (E), and ACH-000856 (F). The center of the figure represents the drug epothilone B, the inner ring represents the top 10 pathways, and the outer ring represents the genes ranked top 25%. The size of the ring indicates the importance of the gene, which is evaluated by the absolute value of the weight extracted from the sparse linear layer of cell line branch.

We assessed the metaDRP model’s predictive accuracy and generalization capabilities using PDTX and TCGA datasets. Using 976 drug-model pairs from breast cancer patients in the PDTX dataset [31], we compared 0-shot and 5-shot learning performance across 75 drugs. The overall performance (SCC^t^) of 5-shot metaDRP in breast cancer tissue across different drugs was 0.5397, a significant improvement over the 0-shot SCC^t^ of 0.3828 (Fig. S2F). Notably, 60% (45/75) of drugs showed enhanced predictive accuracy, with 7 drugs achieving SCC^d^ > 0.5 (Figs. 4A and B).

Evaluation in the TCGA dataset further validated the model’s inductive reasoning capabilities. Seven drugs achieved predicted ROC_AUC greater than 0.5 (Fig. 4C), with cisplatin showing significant responder/non-responder discrimination (effect size >0, *P* value < 0.05). 0-shot metaDRP outperformed MOLI and DrugCell but was slightly inferior to TGSA and PaccMann (Table S11). Specifically, the prediction of metaDRP for sorafenib was the highest among all methods, with a ROC_AUC of 0.846. 10-shot fine-tuning improved ROC_AUC for 6 out of 9 drugs, outperforming benchmarks in cisplatin and pemetrexed. These findings highlight the potential of metaDRP for clinical translation, where minimal preclinical samples can significantly enhance prediction transferability to *in vivo* patient settings.

### 3.8 Model explainer analyzes the MoAs of triple negative breast cancer (TNBC) cell lines with epothilone B

Using the metaDRP model, we evaluated the efficacy of FDA-approved and investigational breast cancer drugs (e.g., epothilone B, gemcitabine, apitolisib, veliparib) on TNBC cell lines. Our analysis leveraged the model’s interpretability framework to dissect pathway and gene importance for top-performing compounds, providing mechanistic insights into their actions. Pathway ranking and gene prioritization enabled by the explainer visualized drug-target interactions within TNBC cell lines. This process identified the top 10 pathways and subsequently isolated genes with absolute weights in the top 25% within these pathways.

Epothilone B, a microtubule-stabilizing agent, binds αβ-tubulin heterodimer to reduce dissociation, inducing G2-M cell cycle arrest and apoptosis. It is FDA-approved as ixabepilone for advanced breast cancer treatment and is beneficial in combination with gemcitabine following the failure of primary therapies [38]. Our analysis emphasized epothilone B’s impact on microtubule-associated proteins and corresponding pathways essential for its cytotoxic effects. Notably, 5 out of 10 TNBC cell line samples (ACH-000212, ACH-000621, ACH-000288, ACH-000643 and ACH-000856) successfully identified drug-targeted microtubule proteins (Figs. 4D–F, Table S12, and Text S2). Similar analyses for other three drugs (gemcitabine, apitolisib, and veliparib) were detailed in the Texts S3 and S4. These findings further validate the explainer model’s capability to map the molecular impact of these drugs within TNBC cell lines, enhancing our understanding of their potential therapeutic benefits in specific cancer contexts.

### 3.9 Evaluating the interpretability of metaDRP in clinical cancer datasets

Our subsequent investigation focused on whether the model’s intrinsic interpretability could be applied to *in vivo* data. We analyzed drug response-annotated samples from the TCGA dataset and selected pemetrexed for further analysis due to its improved ROC_AUC performance after few-shot fine-tuning. Pemetrexed, a multi-target antifolate agent, has exhibited broad-spectrum antitumor activity in various Phase II clinical trials for a range of solid tumors, including non-small cell lung cancer and malignant mesothelioma [39].

Interpretability analysis of 8 lung adenocarcinoma (LUAD) samples from the TCGA dataset revealed that in the 0-shot setting, three samples (TCGA-91-7771, TCGA-L9-A7SV, TCGA-64-5779) successfully identified DHFR, the known target of pemetrexed, along with its associated pathway folate biosynthesis (Table S13). Comparative analysis between 0-shot and 10-shot settings showed that while 10-shot learning improved ROC_AUC scores, it reduced interpretability as only 3/8 samples identified correct target pathways, with TCGA-64-5779 being the sole sample recognizing the drug target. Analysis of 50 LUAD cell line samples demonstrated strong 0-shot interpretability, with 31/50 samples correctly identifying the drug target (Table S14). This highlights the potential reliability of metaDRP capturing drug-pathway relationships effectively even without fine-tuning in decision-making for patients. 10-shot fine-tuning could further improve metaDRP’s interpretability on cell line samples, with 32 of these samples identified with the correct drug target (Table S14). This observation highlights a key difference between clinical and preclinical applications of the model: while few-shot with preclinical samples improves interpretability in cell line data, it exhibits deficiencies in clinical datasets.

## 4 Conclusion and discussion

In this work, we developed metaDRP, an interpretable deep learning framework designed for few-shot drug response prediction. Our findings demonstrate the robustness of metaDRP across diverse datasets and predictive tasks, particularly in low-data environments. The framework’s success stems from three synergistic innovations: (1) Pretrained drug encoder enables cross-modal knowledge transfer; (2) MoA-informed attention mechanisms capture pathway-specific signatures; (3) Few-shot learning architecture facilitates rapid adaptation to new tasks with minimal data. The challenge posed by OOD discrepancies across clinical cohorts has historically hindered the widespread adoption of DRP models. The metaDRP’s application to external datasets such as TCGA and PDTX highlights its potential for real-world clinical translation, demonstrating actionable insights for treatment decisions. Notably, the model’s capability of leveraging preclinical data for fine-tuning further supports its utility in clinical decision-making, as evidenced by its improved prediction performance for drugs like pemetrexed and cisplatin in TCGA dataset. While demonstrating cross-domain adaptability, key limitations emerged in tissue microenvironment modeling and loss function alignment with predictive accuracy. Despite these challenges, our study pioneers the use of few-shot learning to bridge preclinical-clinical data gaps, addressing longstanding issues of data scarcity and OOD generalization. Overall, metaDRP’s dual capability to provide both predictive accuracy and biological interpretability positions it as a potential tool in drug development and personalized medicine.

## CRediT authorship contribution statement

**Hui Guo:** Writing – original draft, Visualization, Validation, Software, Methodology, Investigation, Formal analysis, Data curation, Conceptualization. **Xiang Lv:** Validation, Software, Methodology, Data curation. **Shenghao Li:** Writing – review & editing, Visualization, Validation, Data curation. **Daichuan Ma:** Funding acquisition, Conceptualization. **Yizhou Li:** Writing – review & editing, Supervision, Resources, Project administration, Methodology, Conceptualization. **Menglong Li:** Writing – review & editing, Resources, Project administration, Funding acquisition, Conceptualization.

## Data availability

Multi-omics molecular profiles of cancer cell lines were acquired from DepMap dataset (https://depmap.org/portal/download/). GDSCv1 drug screening dataset was downloaded from CellMinerCDB [29] and the TNBC cell lines was annotated according to Dai et al. [26]. ZINC 250k dataset for pretraining the drug encoder was obtained from Chemical VAE [30]. PDX data was downloaded from Chen et al. [31] and TCGA patient gene expression data was from Ding et al. [32].

## Code availability

The models were implemented using Python (v 3.9.12) and Pytorch (v 1.12.1). The code for metaDRP is publicly available at https://github.com/cicGuoH/metaDRP.

## Declaration of generative AI in scientific writing

During the preparation of this work the authors used DeepSeek in order to improve readability and language. After using this tool, the authors reviewed and edited the content as needed and take full responsibility for the content of the publication.

## Declaration of competing interest

The authors declare that there are no conflicts of interest.
