# Supplementary material for: Fast-adapting graph neural network with prior knowledge for drug response prediction across preclinical and clinical data
Source: J Pharm Anal. 2025 Jul 4;15(10):101386. doi: 10.1016/j.jpha.2025.101386 (PMC12613006; doi:10.1016/j.jpha.2025.101386)
Supplement: Multimedia component 2 [file mmc2.docx]

# Figure S1


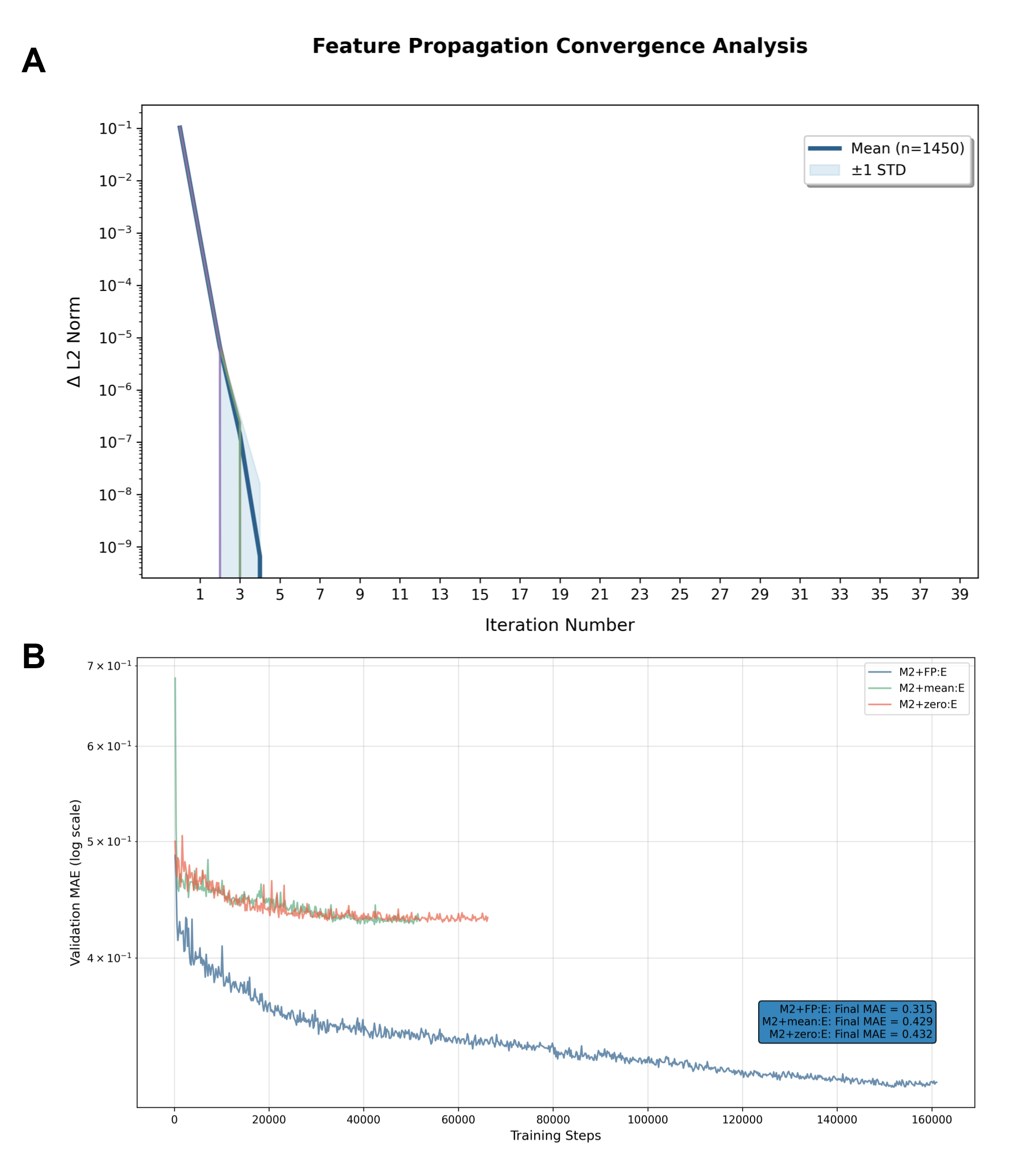


**Fig. S1.** (A) Visualization of $\Delta$L2 norm differences (log scale) across 40 iterations for 1,450 samples using feature imputation. Semi-transparent blue traces represent individual samples, with the bold blue line indicating population mean±1 standard deviation (shaded region). All samples converged around iteration 4. (B) Validation loss (Mean Square Error, MSE) trajectories (log scale) for three imputation strategies (M2+FP: feature propagation, M2+zero: zero-filling, M2+mean: mean imputation).

# Figure S2


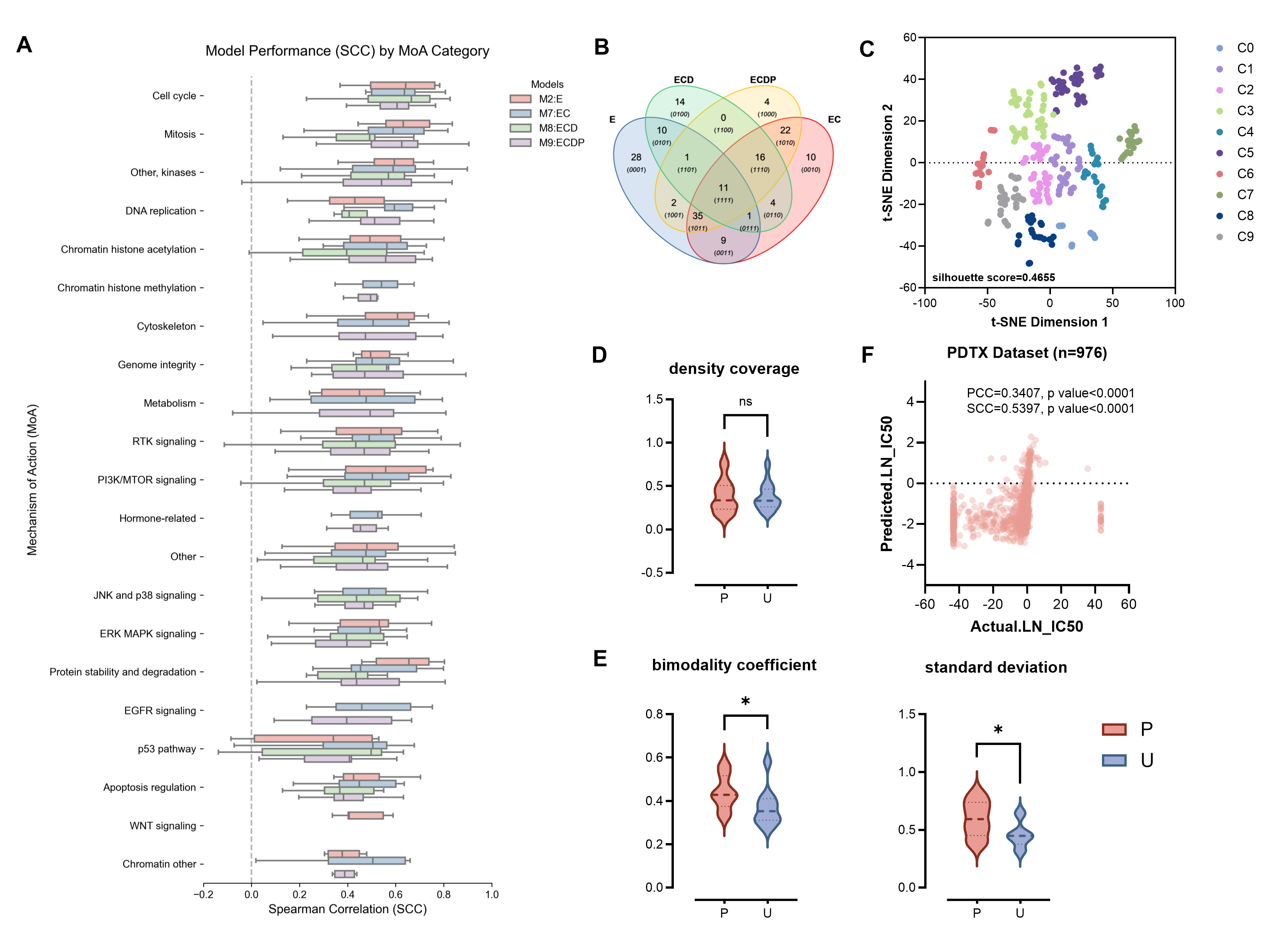


**Fig. S2.** (A) Mechanism of action (MoA)-specific model performance. Boxplot illustrates multi-modal fusion model performance (SCC^dt^) by MoA categories. (B) Upset plot depicting overlaps in highly predictive drugs (SCC^d^ > 0.5) across four multi-omics configurations: E, EC, ECD and ECDP (E: gene expression, C: copy number variations, D: DNA methylation, P: CRISPR-Cas9 gene effects). (C) *t*-SNE visualization of drug embeddings obtained from non-pretrained drug encoder colored by k-Means clusters. (D) Violin plot displaying the distribution of density coverage for predictable (P-class) and unpredictable (U-class) drugs. (E) Violin plots of bimodality coefficient and standard deviation for P/U-class drugs in Unseen Drug dataset. (F) PDTX Dataset Validation. Scatter plot compares predicted vs. actual LN_IC50 values for breast cancer PDX models.

# Table S1. Main Hyperparameter setting of metaDRP.

| **Drug branch** | |
| --- | --- |
| drug_max_nodes | 64 |
| spatial_pos_max | 64 |
| drug_num_atoms | 64*9 |
| drug_num_in_degree | 64 |
| drug_num_out_degree | 64 |
| drug_num_edges | 64*3 |
| drug_num_spatial | 64 |
| drug_num_edge_dist | 5 |
| drug_multi_hop_max_dist | 5 |
| drug_n_encode_layers | 12 |
| drug_embed_dim | 80 |
| drug_num_heads | 8 |
| **Cell line branch** | |
| num_genes | 4446 |
| in_features | 1 |
| num_pathways | 186 |
| ccl_num_heads | 8 |
| num_pattn_layers | 1 |
| embed_dim | 80 |
| dropout_rate | 0.1 |
| cd_num_heads | 16 |
| ccl_drug_trans | True |
| **Training parameters** |  |
| meta_lr | 1e-4 |
| inner_lr | 1e-4 |
| total_epochs | 1000 |
| pretrain_flag | True |
| patience | 50 |
| lr_scheduler_name | cosine |
| decay_rate | 0.1 |
| warmup_lr | 8e-5 |
| min_lr | 1e-5 |
| warmup_epochs | 40 |
| decay_epochs | 40 |

# Table S2. Hyperparameter optimization of baseline ML methods.

| Model | Parameter | Values | #combinations |
| --- | --- | --- | --- |
| SVR | C | $2^{v}$:$v\in$ [-10, 10] in steps of 2 | 22 |
|  | kernel | [linear, rbf] |  |
| RF | n_estimators | [500, 1000, 1500] | 9 |
|  | max_features | [5, 55,105] |  |
| Ridge | alpha | 21 equally spaced values $\in$ [0,1] | 21 |

#combinations: number of combinations; Ridge: ridge regression; RF: random forest; SVR: support vector machine.

# Text S1. The effective fusion of multi-omics features

The integration of CNV data into the M7 model improved SCC^d^ for 52.98% of the drugs compared with M2 (Table S4), with significant increases noted in drugs such as NSC-207895, a MDM4 inhibitor, highlighting the predictive relevance of CNVs in tumor suppressor pathways[1]. Upon comparing M2 and M8 (Tables S5 and S7) it was observed that 34.02% (33/97) of the drugs exhibited an enhancement in SCC^d^ when incorporating methylation features alongside gene expression and CNVs in M8. Nutlin-3a, a small molecule antagonist of the p53/MDM2 pathway, saw a notable improvement in M7 and M8 compared to M2 indicating that copy number variation features and methylation epigenetic features are linked to Nutlin-3a response patterns. It was reported that specific p53 mutations are associated with copy number variations in studies on ovarian cancer[2]. Additionally, research has shown that the entire gene expression profile and epigenetic status are effective biomarkers of sensitivity to Nutlin-3a in the treatment of malignant tumors [3], further validating the effective fusion of multi-omics features in our model.

# Text S2. Explainer can identify the correct target for TNBC cell lines with drug epothilone B

target pathways: GAP_JUNCTION, APOPTOSIS, CELL_CYCLE

target genes: TUBA3C, TUBB, TUBB1, TUBB4B, TUBB4A, TUBA4A, TUBB3, TUBA1C, TUBA8, TUBA1B, TUBA1A

## Identification of key genes and pathways for TNBC cell line ACH-000856.


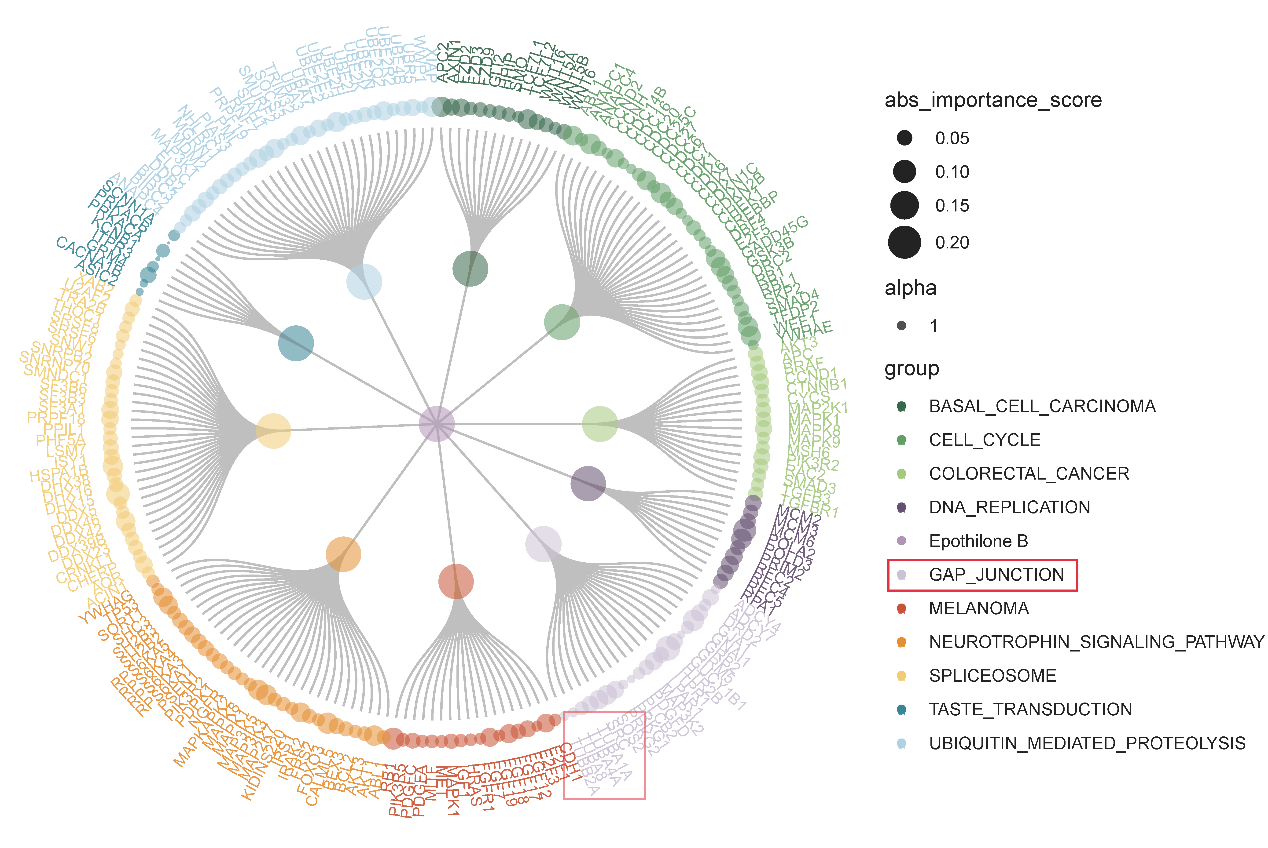


| **name** | **type** | **score** | **raw_name** |
| --- | --- | --- | --- |
| **GAP_JUNCTION** | **Pathway** | **1.20603168** | **GAP_JUNCTION** |
| COLORECTAL_CANCER | Pathway | 1.191164374 | COLORECTAL_CANCER |
| **CELL_CYCLE** | **Pathway** | **1.190423489** | **CELL_CYCLE** |
| UBIQUITIN_MEDIATED_PROTEOLYSIS | Pathway | 1.17739296 | UBIQUITIN_MEDIATED_PROTEOLYSIS |
| TASTE_TRANSDUCTION | Pathway | 1.174840808 | TASTE_TRANSDUCTION |
| BASAL_CELL_CARCINOMA | Pathway | 1.173064113 | BASAL_CELL_CARCINOMA |
| SPLICEOSOME | Pathway | 1.154335022 | SPLICEOSOME |
| NEUROTROPHIN_SIGNALING_PATHWAY | Pathway | 1.153535247 | NEUROTROPHIN_SIGNALING_PATHWAY |
| MELANOMA | Pathway | 1.152503848 | MELANOMA |
| DNA_REPLICATION | Pathway | 1.143480897 | DNA_REPLICATION |
| Epothilone B | Drug | 1.190119505 | Epothilone B |
| KEGG_CELL_CYCLE-ABL1 | Gene | -0.074554816 | ABL1 |
| KEGG_CELL_CYCLE-ANAPC1 | Gene | -0.039530326 | ANAPC1 |
| KEGG_CELL_CYCLE-ANAPC4 | Gene | -0.08301834 | ANAPC4 |
| KEGG_CELL_CYCLE-CCND2 | Gene | -0.051464241 | CCND2 |
| KEGG_CELL_CYCLE-CCNH | Gene | 0.032268401 | CCNH |
| KEGG_CELL_CYCLE-CDC14B | Gene | -0.066123776 | CDC14B |
| KEGG_CELL_CYCLE-CDC16 | Gene | 0.032459036 | CDC16 |
| KEGG_CELL_CYCLE-CDC20 | Gene | -0.030078385 | CDC20 |
| KEGG_CELL_CYCLE-CDC25C | Gene | -0.042172737 | CDC25C |
| KEGG_CELL_CYCLE-CDC27 | Gene | -0.073725283 | CDC27 |
| KEGG_CELL_CYCLE-CDC6 | Gene | -0.039519031 | CDC6 |
| KEGG_CELL_CYCLE-CDC7 | Gene | -0.079953998 | CDC7 |
| KEGG_CELL_CYCLE-CDK1 | Gene | -0.05435868 | CDK1 |
| KEGG_CELL_CYCLE-CDK6 | Gene | 0.058156896 | CDK6 |
| KEGG_CELL_CYCLE-CDK7 | Gene | 0.031918682 | CDK7 |
| KEGG_CELL_CYCLE-CDKN1C | Gene | 0.030728221 | CDKN1C |
| KEGG_CELL_CYCLE-CDKN2B | Gene | 0.047359932 | CDKN2B |
| KEGG_CELL_CYCLE-CHEK1 | Gene | -0.035832919 | CHEK1 |
| KEGG_CELL_CYCLE-CREBBP | Gene | -0.037866622 | CREBBP |
| KEGG_CELL_CYCLE-DBF4 | Gene | 0.034663983 | DBF4 |
| KEGG_CELL_CYCLE-E2F5 | Gene | -0.074107349 | E2F5 |
| KEGG_CELL_CYCLE-GADD45G | Gene | 0.076305524 | GADD45G |
| KEGG_CELL_CYCLE-GSK3B | Gene | -0.062222946 | GSK3B |
| KEGG_CELL_CYCLE-ORC2 | Gene | -0.05312233 | ORC2 |
| KEGG_CELL_CYCLE-RB1 | Gene | -0.043507461 | RB1 |
| KEGG_CELL_CYCLE-RBL1 | Gene | -0.034489203 | RBL1 |
| KEGG_CELL_CYCLE-SKP2 | Gene | 0.048041575 | SKP2 |
| KEGG_CELL_CYCLE-SMAD4 | Gene | -0.041809645 | SMAD4 |
| KEGG_CELL_CYCLE-TFDP2 | Gene | 0.079277799 | TFDP2 |
| KEGG_CELL_CYCLE-WEE1 | Gene | -0.077003896 | WEE1 |
| KEGG_CELL_CYCLE-YWHAE | Gene | -0.030563986 | YWHAE |
| KEGG_GAP_JUNCTION-ADCY4 | Gene | -0.028222868 | ADCY4 |
| KEGG_GAP_JUNCTION-ADCY7 | Gene | 0.056999445 | ADCY7 |
| KEGG_GAP_JUNCTION-DRD2 | Gene | -0.036155038 | DRD2 |
| KEGG_GAP_JUNCTION-GJA1 | Gene | 0.058210772 | GJA1 |
| KEGG_GAP_JUNCTION-GNAI1 | Gene | -0.08704333 | GNAI1 |
| KEGG_GAP_JUNCTION-GRB2 | Gene | -0.040112562 | GRB2 |
| KEGG_GAP_JUNCTION-GRM5 | Gene | -0.024476334 | GRM5 |
| KEGG_GAP_JUNCTION-GUCY1B1 | Gene | -0.056147434 | GUCY1B1 |
| KEGG_GAP_JUNCTION-HTR2B | Gene | -0.113540784 | HTR2B |
| KEGG_GAP_JUNCTION-ITPR1 | Gene | 0.052234273 | ITPR1 |
| KEGG_GAP_JUNCTION-LPAR1 | Gene | -0.064293265 | LPAR1 |
| KEGG_GAP_JUNCTION-MAP3K2 | Gene | -0.05098512 | MAP3K2 |
| KEGG_GAP_JUNCTION-PDGFD | Gene | -0.037310518 | PDGFD |
| KEGG_GAP_JUNCTION-PLCB2 | Gene | -0.024488829 | PLCB2 |
| KEGG_GAP_JUNCTION-PRKG1 | Gene | 0.028834615 | PRKG1 |
| KEGG_GAP_JUNCTION-PRKG2 | Gene | -0.062303543 | PRKG2 |
| KEGG_GAP_JUNCTION-SOS2 | Gene | -0.075955421 | SOS2 |
| KEGG_GAP_JUNCTION-SRC | Gene | -0.07215178 | SRC |
| **KEGG_GAP_JUNCTION-TUBA1A** | **Gene** | **-0.057586648** | **TUBA1A** |
| **KEGG_GAP_JUNCTION-TUBA4A** | **Gene** | **-0.04367226** | **TUBA4A** |
| **KEGG_GAP_JUNCTION-TUBA8** | **Gene** | **-0.029078837** | **TUBA8** |
| **KEGG_GAP_JUNCTION-TUBB2A** | **Gene** | **-0.028069237** | **TUBB2A** |

## 2.2 Identification of key genes and pathways for TNBC cell line ACH-000212


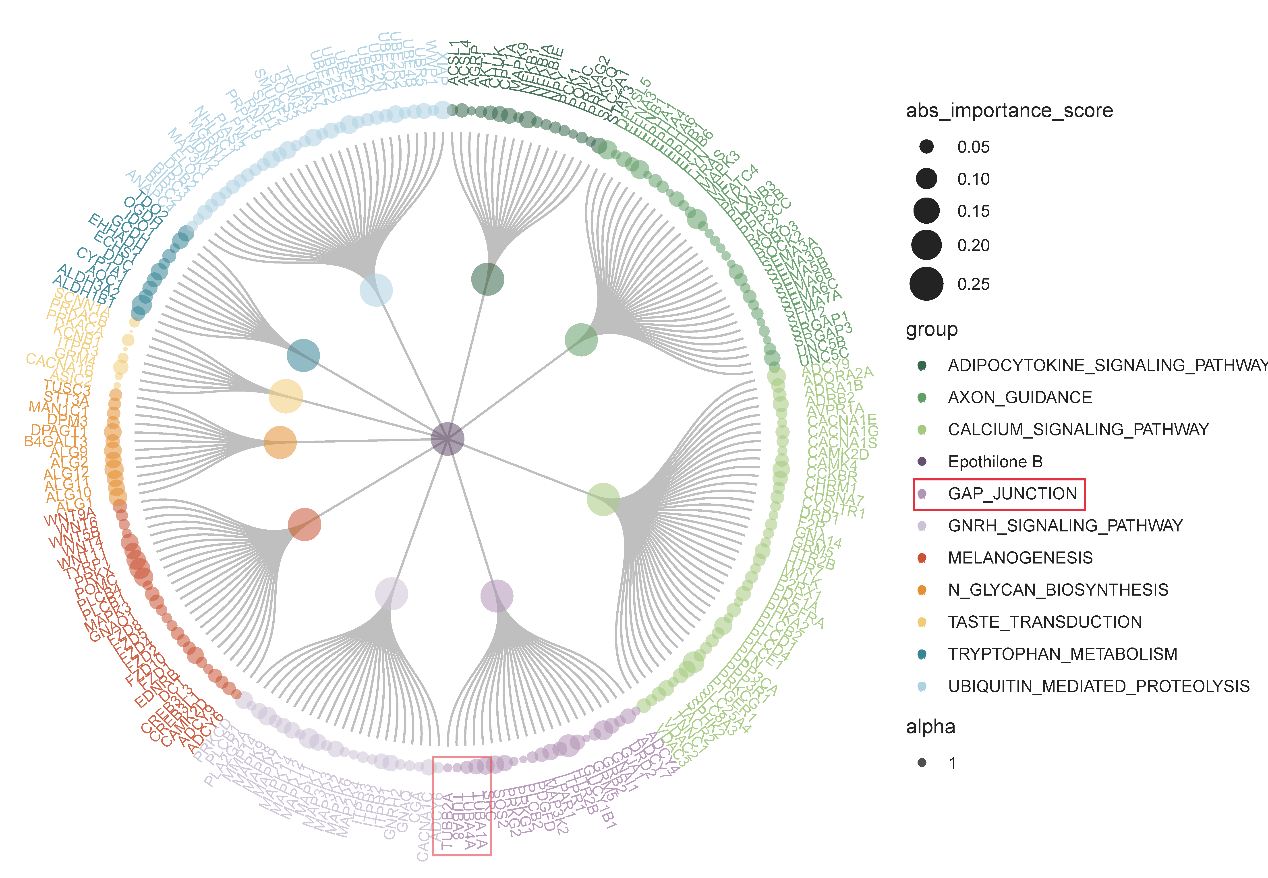


| **name** | **type** | **score** | **raw_name** |
| --- | --- | --- | --- |
| TASTE_TRANSDUCTION | Pathway | 1.297968 | TASTE_TRANSDUCTION |
| CALCIUM_SIGNALING_PATHWAY | Pathway | 1.200434 | CALCIUM_SIGNALING_PATHWAY |
| UBIQUITIN_MEDIATED_PROTEOLYSIS | Pathway | 1.196775 | UBIQUITIN_MEDIATED_PROTEOLYSIS |
| MELANOGENESIS | Pathway | 1.196749 | MELANOGENESIS |
| TRYPTOPHAN_METABOLISM | Pathway | 1.189761 | TRYPTOPHAN_METABOLISM |
| AXON_GUIDANCE | Pathway | 1.186952 | AXON_GUIDANCE |
| GNRH_SIGNALING_PATHWAY | Pathway | 1.185304 | GNRH_SIGNALING_PATHWAY |
| **GAP_JUNCTION** | **Pathway** | **1.183033** | **GAP_JUNCTION** |
| N_GLYCAN_BIOSYNTHESIS | Pathway | 1.182365 | N_GLYCAN_BIOSYNTHESIS |
| ADIPOCYTOKINE_SIGNALING_PATHWAY | Pathway | 1.181111 | ADIPOCYTOKINE_SIGNALING_PATHWAY |
| Epothilone B | Drug | 1.225707 | Epothilone B |
| KEGG_GAP_JUNCTION-ADCY4 | Gene | -0.02822 | ADCY4 |
| KEGG_GAP_JUNCTION-ADCY7 | Gene | 0.056999 | ADCY7 |
| KEGG_GAP_JUNCTION-DRD2 | Gene | -0.03616 | DRD2 |
| KEGG_GAP_JUNCTION-GJA1 | Gene | 0.058211 | GJA1 |
| KEGG_GAP_JUNCTION-GNAI1 | Gene | -0.08704 | GNAI1 |
| KEGG_GAP_JUNCTION-GRB2 | Gene | -0.04011 | GRB2 |
| KEGG_GAP_JUNCTION-GRM5 | Gene | -0.02448 | GRM5 |
| KEGG_GAP_JUNCTION-GUCY1B1 | Gene | -0.05615 | GUCY1B1 |
| KEGG_GAP_JUNCTION-HTR2B | Gene | -0.11354 | HTR2B |
| KEGG_GAP_JUNCTION-ITPR1 | Gene | 0.052234 | ITPR1 |
| KEGG_GAP_JUNCTION-LPAR1 | Gene | -0.06429 | LPAR1 |
| KEGG_GAP_JUNCTION-MAP3K2 | Gene | -0.05099 | MAP3K2 |
| KEGG_GAP_JUNCTION-PDGFD | Gene | -0.03731 | PDGFD |
| KEGG_GAP_JUNCTION-PLCB2 | Gene | -0.02449 | PLCB2 |
| KEGG_GAP_JUNCTION-PRKG1 | Gene | 0.028835 | PRKG1 |
| KEGG_GAP_JUNCTION-PRKG2 | Gene | -0.0623 | PRKG2 |
| KEGG_GAP_JUNCTION-SOS2 | Gene | -0.07596 | SOS2 |
| KEGG_GAP_JUNCTION-SRC | Gene | -0.07215 | SRC |
| **KEGG_GAP_JUNCTION-TUBA1A** | **Gene** | **-0.05759** | **TUBA1A** |
| **KEGG_GAP_JUNCTION-TUBA4A** | **Gene** | **-0.04367** | **TUBA4A** |
| **KEGG_GAP_JUNCTION-TUBA8** | **Gene** | **-0.02908** | **TUBA8** |
| **KEGG_GAP_JUNCTION-TUBB2A** | **Gene** | **-0.02807** | **TUBB2A** |

## 2.3 Identification of key genes and pathways for TNBC cell line ACH-000621


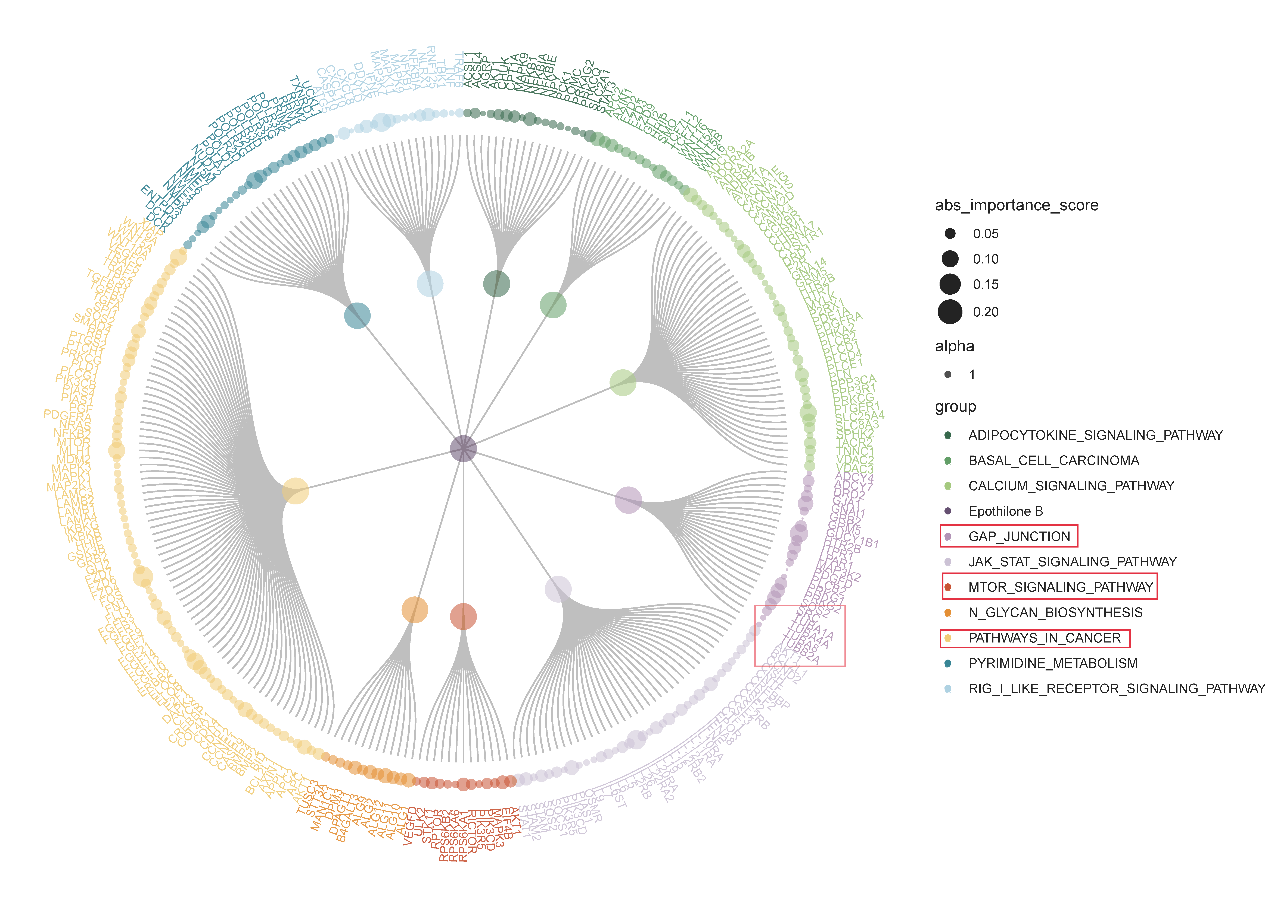


| **name** | **type** | **score** | **raw_name** |
| --- | --- | --- | --- |
| **GAP_JUNCTION** | **Pathway** | **1.211935** | **GAP_JUNCTION** |
| PATHWAYS_IN_CANCER | Pathway | 1.185587 | PATHWAYS_IN_CANCER |
| ADIPOCYTOKINE_SIGNALING_PATHWAY | Pathway | 1.18122 | ADIPOCYTOKINE_SIGNALING_PATHWAY |
| JAK_STAT_SIGNALING_PATHWAY | Pathway | 1.174338 | JAK_STAT_SIGNALING_PATHWAY |
| CALCIUM_SIGNALING_PATHWAY | Pathway | 1.172678 | CALCIUM_SIGNALING_PATHWAY |
| MTOR_SIGNALING_PATHWAY | Pathway | 1.160022 | MTOR_SIGNALING_PATHWAY |
| BASAL_CELL_CARCINOMA | Pathway | 1.159988 | BASAL_CELL_CARCINOMA |
| N_GLYCAN_BIOSYNTHESIS | Pathway | 1.159673 | N_GLYCAN_BIOSYNTHESIS |
| RIG_I_LIKE_RECEPTOR_SIGNALING_PATHWAY | Pathway | 1.154374 | RIG_I_LIKE_RECEPTOR_SIGNALING_PATHWAY |
| PYRIMIDINE_METABOLISM | Pathway | 1.147359 | PYRIMIDINE_METABOLISM |
| Epothilone B | Drug | 1.178354 | Epothilone B |
| KEGG_GAP_JUNCTION-ADCY4 | Gene | -0.02822 | ADCY4 |
| KEGG_GAP_JUNCTION-ADCY7 | Gene | 0.056999 | ADCY7 |
| KEGG_GAP_JUNCTION-DRD2 | Gene | -0.03616 | DRD2 |
| KEGG_GAP_JUNCTION-GJA1 | Gene | 0.058211 | GJA1 |
| KEGG_GAP_JUNCTION-GNAI1 | Gene | -0.08704 | GNAI1 |
| KEGG_GAP_JUNCTION-GRB2 | Gene | -0.04011 | GRB2 |
| KEGG_GAP_JUNCTION-GRM5 | Gene | -0.02448 | GRM5 |
| KEGG_GAP_JUNCTION-GUCY1B1 | Gene | -0.05615 | GUCY1B1 |
| KEGG_GAP_JUNCTION-HTR2B | Gene | -0.11354 | HTR2B |
| KEGG_GAP_JUNCTION-ITPR1 | Gene | 0.052234 | ITPR1 |
| KEGG_GAP_JUNCTION-LPAR1 | Gene | -0.06429 | LPAR1 |
| KEGG_GAP_JUNCTION-MAP3K2 | Gene | -0.05099 | MAP3K2 |
| KEGG_GAP_JUNCTION-PDGFD | Gene | -0.03731 | PDGFD |
| KEGG_GAP_JUNCTION-PLCB2 | Gene | -0.02449 | PLCB2 |
| KEGG_GAP_JUNCTION-PRKG1 | Gene | 0.028835 | PRKG1 |
| KEGG_GAP_JUNCTION-PRKG2 | Gene | -0.0623 | PRKG2 |
| KEGG_GAP_JUNCTION-SOS2 | Gene | -0.07596 | SOS2 |
| KEGG_GAP_JUNCTION-SRC | Gene | -0.07215 | SRC |
| **KEGG_GAP_JUNCTION-TUBA1A** | **Gene** | **-0.05759** | **TUBA1A** |
| **KEGG_GAP_JUNCTION-TUBA4A** | **Gene** | **-0.04367** | **TUBA4A** |
| **KEGG_GAP_JUNCTION-TUBA8** | **Gene** | **-0.02908** | **TUBA8** |
| **KEGG_GAP_JUNCTION-TUBB2A** | **Gene** | **-0.02807** | **TUBB2A** |

## Identification of key genes and pathways for TNBC cell line ACH-000288


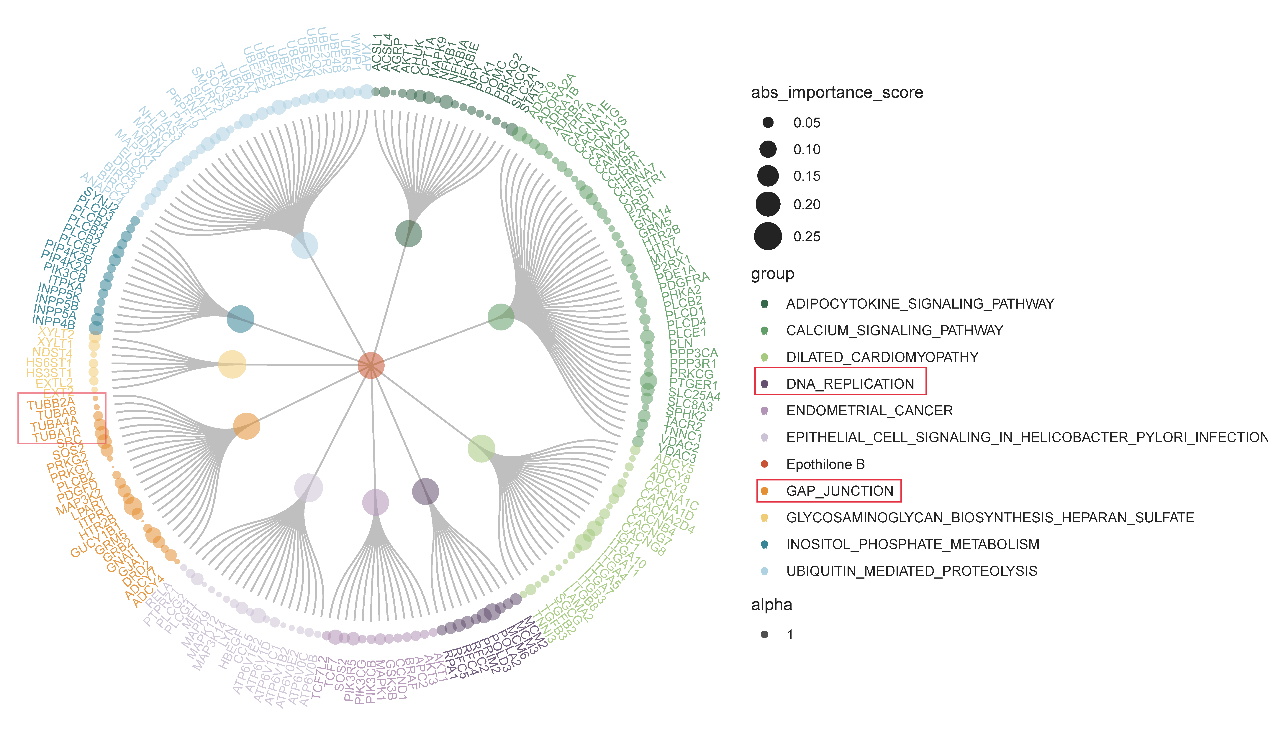


| **name** | **type** | **score** | **raw_name** |
| --- | --- | --- | --- |
| EPITHELIAL_CELL_SIGNALING_IN_HELICOBACTER_PYLORI_INFECTION | Pathway | 1.327167 | EPITHELIAL_CELL_SIGNALING_IN_HELICOBACTER_PYLORI_INFECTION |
| GLYCOSAMINOGLYCAN_BIOSYNTHESIS_HEPARAN_SULFATE | Pathway | 1.292997 | GLYCOSAMINOGLYCAN_BIOSYNTHESIS_HEPARAN_SULFATE |
| DILATED_CARDIOMYOPATHY | Pathway | 1.228926 | DILATED_CARDIOMYOPATHY |
| INOSITOL_PHOSPHATE_METABOLISM | Pathway | 1.210939 | INOSITOL_PHOSPHATE_METABOLISM |
| **GAP_JUNCTION** | **Pathway** | **1.174033** | **GAP_JUNCTION** |
| DNA_REPLICATION | Pathway | 1.16619 | DNA_REPLICATION |
| CALCIUM_SIGNALING_PATHWAY | Pathway | 1.165317 | CALCIUM_SIGNALING_PATHWAY |
| ADIPOCYTOKINE_SIGNALING_PATHWAY | Pathway | 1.15473 | ADIPOCYTOKINE_SIGNALING_PATHWAY |
| UBIQUITIN_MEDIATED_PROTEOLYSIS | Pathway | 1.151561 | UBIQUITIN_MEDIATED_PROTEOLYSIS |
| ENDOMETRIAL_CANCER | Pathway | 1.149116 | ENDOMETRIAL_CANCER |
| Epothilone B | Drug | 1.16106 | Epothilone B |
| KEGG_GAP_JUNCTION-ADCY4 | Gene | -0.02822 | ADCY4 |
| KEGG_GAP_JUNCTION-ADCY7 | Gene | 0.056999 | ADCY7 |
| KEGG_GAP_JUNCTION-DRD2 | Gene | -0.03616 | DRD2 |
| KEGG_GAP_JUNCTION-GJA1 | Gene | 0.058211 | GJA1 |
| KEGG_GAP_JUNCTION-GNAI1 | Gene | -0.08704 | GNAI1 |
| KEGG_GAP_JUNCTION-GRB2 | Gene | -0.04011 | GRB2 |
| KEGG_GAP_JUNCTION-GRM5 | Gene | -0.02448 | GRM5 |
| KEGG_GAP_JUNCTION-GUCY1B1 | Gene | -0.05615 | GUCY1B1 |
| KEGG_GAP_JUNCTION-HTR2B | Gene | -0.11354 | HTR2B |
| KEGG_GAP_JUNCTION-ITPR1 | Gene | 0.052234 | ITPR1 |
| KEGG_GAP_JUNCTION-LPAR1 | Gene | -0.06429 | LPAR1 |
| KEGG_GAP_JUNCTION-MAP3K2 | Gene | -0.05099 | MAP3K2 |
| KEGG_GAP_JUNCTION-PDGFD | Gene | -0.03731 | PDGFD |
| KEGG_GAP_JUNCTION-PLCB2 | Gene | -0.02449 | PLCB2 |
| KEGG_GAP_JUNCTION-PRKG1 | Gene | 0.028835 | PRKG1 |
| KEGG_GAP_JUNCTION-PRKG2 | Gene | -0.0623 | PRKG2 |
| KEGG_GAP_JUNCTION-SOS2 | Gene | -0.07596 | SOS2 |
| KEGG_GAP_JUNCTION-SRC | Gene | -0.07215 | SRC |
| **KEGG_GAP_JUNCTION-TUBA1A** | **Gene** | **-0.05759** | **TUBA1A** |
| **KEGG_GAP_JUNCTION-TUBA4A** | **Gene** | **-0.04367** | **TUBA4A** |
| **KEGG_GAP_JUNCTION-TUBA8** | **Gene** | **-0.02908** | **TUBA8** |
| **KEGG_GAP_JUNCTION-TUBB2A** | **Gene** | **-0.02807** | **TUBB2A** |

## Identification of key genes and pathways for TNBC cell line ACH-000643


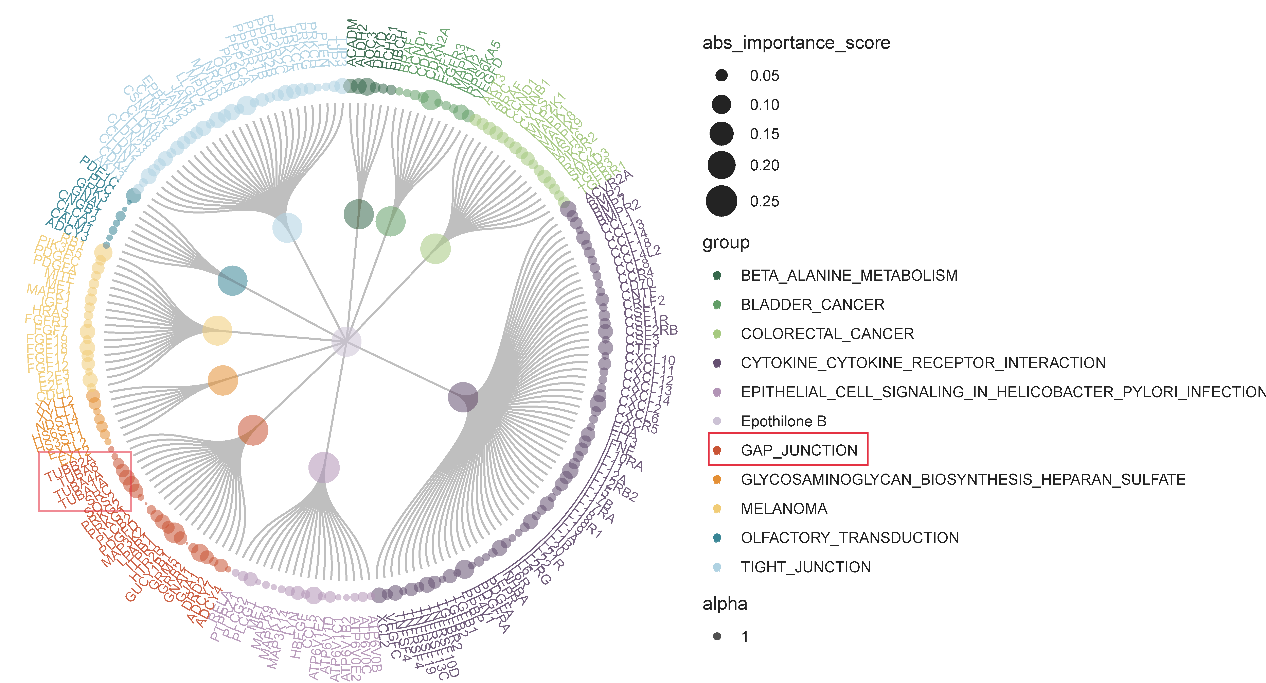


| **name** | **type** | **score** | **raw_name** |
| --- | --- | --- | --- |
| EPITHELIAL_CELL_SIGNALING_IN_HELICOBACTER_PYLORI_INFECTION | Pathway | 1.252417 | EPITHELIAL_CELL_SIGNALING_IN_HELICOBACTER_PYLORI_INFECTION |
| **GAP_JUNCTION** | **Pathway** | **1.196282** | **GAP_JUNCTION** |
| COLORECTAL_CANCER | Pathway | 1.185531 | COLORECTAL_CANCER |
| CYTOKINE_CYTOKINE_RECEPTOR_INTERACTION | Pathway | 1.181854 | CYTOKINE_CYTOKINE_RECEPTOR_INTERACTION |
| GLYCOSAMINOGLYCAN_BIOSYNTHESIS_HEPARAN_SULFATE | Pathway | 1.173075 | GLYCOSAMINOGLYCAN_BIOSYNTHESIS_HEPARAN_SULFATE |
| BLADDER_CANCER | Pathway | 1.162576 | BLADDER_CANCER |
| BETA_ALANINE_METABOLISM | Pathway | 1.155722 | BETA_ALANINE_METABOLISM |
| TIGHT_JUNCTION | Pathway | 1.147837 | TIGHT_JUNCTION |
| MELANOMA | Pathway | 1.141027 | MELANOMA |
| OLFACTORY_TRANSDUCTION | Pathway | 1.140123 | OLFACTORY_TRANSDUCTION |
| Epothilone B | Drug | 1.157264 | Epothilone B |
| KEGG_GAP_JUNCTION-ADCY4 | Gene | -0.02822 | ADCY4 |
| KEGG_GAP_JUNCTION-ADCY7 | Gene | 0.056999 | ADCY7 |
| KEGG_GAP_JUNCTION-DRD2 | Gene | -0.03616 | DRD2 |
| KEGG_GAP_JUNCTION-GJA1 | Gene | 0.058211 | GJA1 |
| KEGG_GAP_JUNCTION-GNAI1 | Gene | -0.08704 | GNAI1 |
| KEGG_GAP_JUNCTION-GRB2 | Gene | -0.04011 | GRB2 |
| KEGG_GAP_JUNCTION-GRM5 | Gene | -0.02448 | GRM5 |
| KEGG_GAP_JUNCTION-GUCY1B1 | Gene | -0.05615 | GUCY1B1 |
| KEGG_GAP_JUNCTION-HTR2B | Gene | -0.11354 | HTR2B |
| KEGG_GAP_JUNCTION-ITPR1 | Gene | 0.052234 | ITPR1 |
| KEGG_GAP_JUNCTION-LPAR1 | Gene | -0.06429 | LPAR1 |
| KEGG_GAP_JUNCTION-MAP3K2 | Gene | -0.05099 | MAP3K2 |
| KEGG_GAP_JUNCTION-PDGFD | Gene | -0.03731 | PDGFD |
| KEGG_GAP_JUNCTION-PLCB2 | Gene | -0.02449 | PLCB2 |
| KEGG_GAP_JUNCTION-PRKG1 | Gene | 0.028835 | PRKG1 |
| KEGG_GAP_JUNCTION-PRKG2 | Gene | -0.0623 | PRKG2 |
| KEGG_GAP_JUNCTION-SOS2 | Gene | -0.07596 | SOS2 |
| KEGG_GAP_JUNCTION-SRC | Gene | -0.07215 | SRC |
| **KEGG_GAP_JUNCTION-TUBA1A** | **Gene** | **-0.05759** | **TUBA1A** |
| **KEGG_GAP_JUNCTION-TUBA4A** | **Gene** | **-0.04367** | **TUBA4A** |
| **KEGG_GAP_JUNCTION-TUBA8** | **Gene** | **-0.02908** | **TUBA8** |
| **KEGG_GAP_JUNCTION-TUBB2A** | **Gene** | **-0.02807** | **TUBB2A** |

# Text S3. Explanations for gemcitabine and TNBC metastatic breast cancer cell line samples

Our analysis extended to Gemcitabine, a potent and selective deoxycytidine analogue known for its antitumor properties. Gemcitabine promotes apoptosis in malignant cells and inhibits DNA synthesis, displaying cytotoxic effects against various in vitro cancer cell lines [4]. It is commonly used as a chemotherapy drug for the first-line treatment of patients with metastatic breast cancer who have failed prior anthracycline-based adjuvant chemotherapy. The explainer’s analysis of TNBC metastatic breast cancer samples, particularly ACH-000212, demonstrated precise identification of the PYRIMIDINE_METABOLISM pathway targeted by Gemcitabine, along with the corresponding RRM1 target. 0-shot meta-learner was more accurate in interpreting Gemcitabine’s MoA, as both ACH-000857 and ACH-000212 were correctly connected with target genes.

**Table A.** Result of target pathway identification with metaDRP using fine-tune approach.

| **item** | **KEGG_PYRIMIDINE_METABOLISM** | **KEGG_PURINE_METABOLISM** |
| --- | --- | --- |
| ACH-000856 | 93 | 15 |
| ACH-000768 | 24 | 111 |
| ACH-000276 | 27 | 39 |
| ACH-000288 | 15 | 66 |
| **ACH-000212** | **9** | 71 |
| ACH-000857 | 30 | 16 |
| ACH-000621 | 43 | 160 |
| ACH-000643 | 88 | 50 |
| ACH-000148 | 45 | 83 |
| ACH-000699 | 81 | 14 |

Using breast cancer cell lines for fine-tuning.

**Table B.** Result of target pathway identification using metaDRP without fine-tuning.

| **item** | **KEGG_PYRIMIDINE_METABOLISM** | **KEGG_PURINE_METABOLISM** |
| --- | --- | --- |
| ACH-000856 | 80 | 12 |
| ACH-000768 | 13 | 101 |
| ACH-000276 | 24 | 33 |
| ACH-000288 | 19 | 47 |
| **ACH-000212** | **8** | 95 |
| **ACH-000857** | **10** | **9** |
| ACH-000621 | 21 | 158 |
| ACH-000643 | 106 | 19 |
| ACH-000148 | 66 | 77 |
| ACH-000699 | 80 | 33 |

Without fine-tuning.

# Text S4. Model explainer analyzes the MoAs of TNBC cell lines with apitolisib and veliparib

Continuing our analysis of targeted therapies, Apitolisib, a PI3K inhibitor, targets the PIK3C and mTOR proteins, primarily affecting the PI3K-Akt signaling pathway and broader cancer pathways[5]. This was reflected in the high rankings for the MTOR_SIGNALING_PATHWAY in samples ACH-000276, ACH-000621, ACH-000768, and ACH-000857, demonstrating the model’s effectiveness in pinpointing relevant therapeutic targets. Similarly, veliparib, a PARP inhibitor, targets PARP1 and PARP2, impacting pathways critical for apoptosis and necroptosis[6]. Notably, the BASE_EXCISION_REPAIR pathway, primarily involving PARP1 and PARP2, was accurately identified in ACH-000856.

**Table A.** Result of target pathway identification for drug apitolisib with metaDRP using fine-tune approach.

| **Apitolisib** | **KEGG_MTOR_SIGNALING_PATHWAY** | **KEGG_PATHWAYS_IN_CANCER** |
| --- | --- | --- |
| ACH-000148 | 116 | 67 |
| ACH-000212 | **20** | 104 |
| ACH-000643 | 67 | 102 |
| ACH-000288 | 48 | 77 |
| ACH-000856 | 69 | 165 |
| ACH-000276 | **13** | 83 |
| ACH-000857 | **16** | 84 |
| ACH-000768 | 187 | 76 |
| ACH-000621 | **18** | 98 |
| ACH-000699 | 63 | 84 |

**Table B.** Result of target pathway identification for drug veliparib with metaDRP using fine-tune approach.

| **Veliparib** | **KEGG_BASE_EXCISION_REPAIR** | **KEGG_APOPTOSIS** |
| --- | --- | --- |
| ACH-000856 | **8** | 52 |
| ACH-000768 | 34 | 103 |
| ACH-000621 | 26 | 98 |
| ACH-000148 | 86 | 105 |
| ACH-000276 | 69 | 66 |
| ACH-000699 | **12** | 65 |
| ACH-000857 | 19 | 82 |
| ACH-000288 | 71 | 97 |
| ACH-000212 | 38 | 94 |
| ACH-000643 | 66 | 82 |

# Reference

[1] O. Karni-Schmidt, M. Lokshin, C. Prives, The Roles of MDM2 and MDMX in Cancer, Annual Review of Pathology: Mechanisms of Disease 11 (2016) 617–644. https://doi.org/10.1146/annurev-pathol-012414-040349.

[2] L.K. Mullany, K.-K. Wong, D.C. Marciano, et al., Specific TP53 Mutants Overrepresented in Ovarian Cancer Impact CNV, TP53 Activity, Responses to Nutlin-3a, and Cell Survival, Neoplasia 17 (2015) 789–803. https://doi.org/10.1016/j.neo.2015.10.003.

[3] K.I. Pishas, S.J. Neuhaus, M.T. Clayer, et al., Nutlin-3a Efficacy in Sarcoma Predicted by Transcriptomic and Epigenetic Profiling, Cancer Research 74 (2014) 921–931. https://doi.org/10.1158/0008-5472.CAN-13-2424.

[4] K. Hastak, E. Alli, J.M. Ford, Synergistic chemosensitivity of triple-negative breast cancer cell lines to PARP inhibition, gemcitabine and cisplatin, Cancer Res 70 (2010) 7970–7980. https://doi.org/10.1158/0008-5472.CAN-09-4521.

[5] S.O. Dolly, A.J. Wagner, J.C. Bendell, et al., Phase I Study of Apitolisib (GDC-0980), Dual Phosphatidylinositol-3-Kinase and Mammalian Target of Rapamycin Kinase Inhibitor, in Patients with Advanced Solid Tumors, Clin. Cancer Res. 22 (2016) 2874–2884. https://doi.org/10.1158/1078-0432.CCR-15-2225.

[6] R.L. Coleman, G.F. Fleming, M.F. Brady, et al., Veliparib with First-Line Chemotherapy and as Maintenance Therapy in Ovarian Cancer, New England Journal of Medicine 381 (2019) 2403–2415. https://doi.org/10.1056/NEJMoa1909707.
